# An optimized transfer learning approach integrating deep convolutional feature extractors for malaria parasite classification in erythrocyte microscopy

**DOI:** 10.3389/fmed.2025.1684973

**Published:** 2025-11-06

**Authors:** C. Kishor Kumar Reddy, P. R. Anisha, Ahlam Almushharaf, Radhika Talla, Jamel Baili, Yongwon Cho, Yunyoung Nam

**Affiliations:** 1 Department of Computer Science and Engineering, Stanley College of Engineering and Technology for Women, Hyderabad, India; 2 Department of Management, College of Business Administration, Princess Nourah bint Abdulrahman University, Riyadh, Saudi Arabia; 3 Department of Computer Engineering, College of Computer Science, King Khalid University, Abha, Saudi Arabia; 4 Department of Computer Science and Engineering, Soonchunhyang University, Asan, Republic of Korea

**Keywords:** malaria diagnosis, transfer learning, automated microscopy, ensemble learning, convolutional neural network

## Abstract

**Background:**

Malaria, caused by *Plasmodium* parasites transmitted through bites from infected female *Anopheles* mosquitoes, results in severe symptoms such as anemia and potential organ failure. The high prevalence of malaria necessitates reliable diagnostic methods to reduce the workload of microscopists, particularly in resource-limited settings.

**Methods:**

This paper evaluates the efficacy of an ensemble learning approach for automated malaria diagnosis. The proposed model integrates convolutional ensemble methods, combining outputs from transfer learning architectures such as VGG16, ResNet50V2, DenseNet201, and VGG19. Data augmentation and pre-processing techniques were applied to enhance robustness, and the ensemble approach was fine-tuned for optimal hyperparameters.

**Results:**

The ensemble achieves a test accuracy of 97.93% by combining a evidence of CNN with multiple transfer learning models (VGG16, ResNet50V2, DenseNet201, and VGG19), with an F1-score and precision of 0.9793 each, outperforming standalone models like Custom CNN (accuracy: 97.20%, F1-score: 0.9720), VGG16 (accuracy: 97.65%, F1-score: 0.9765), and CNN-SVM (accuracy: 82.47%, F1-score: 0.8266). The method demonstrated effectiveness in classifying parasitized and uninfected blood smears with high reliability, addressing the limitations of manual microscopy and standalone models.

**Conclusion:**

The proposed ensemble learning approach highlights the potential of integrating transfer learning models to improve diagnostic accuracy for malaria detection. This scalable, automated solution reduces reliance on manual microscopy, making it highly applicable in resource-constrained settings and offering a significant advancement in malaria diagnostics.

## 1 Introduction

**FIGURE 1:**
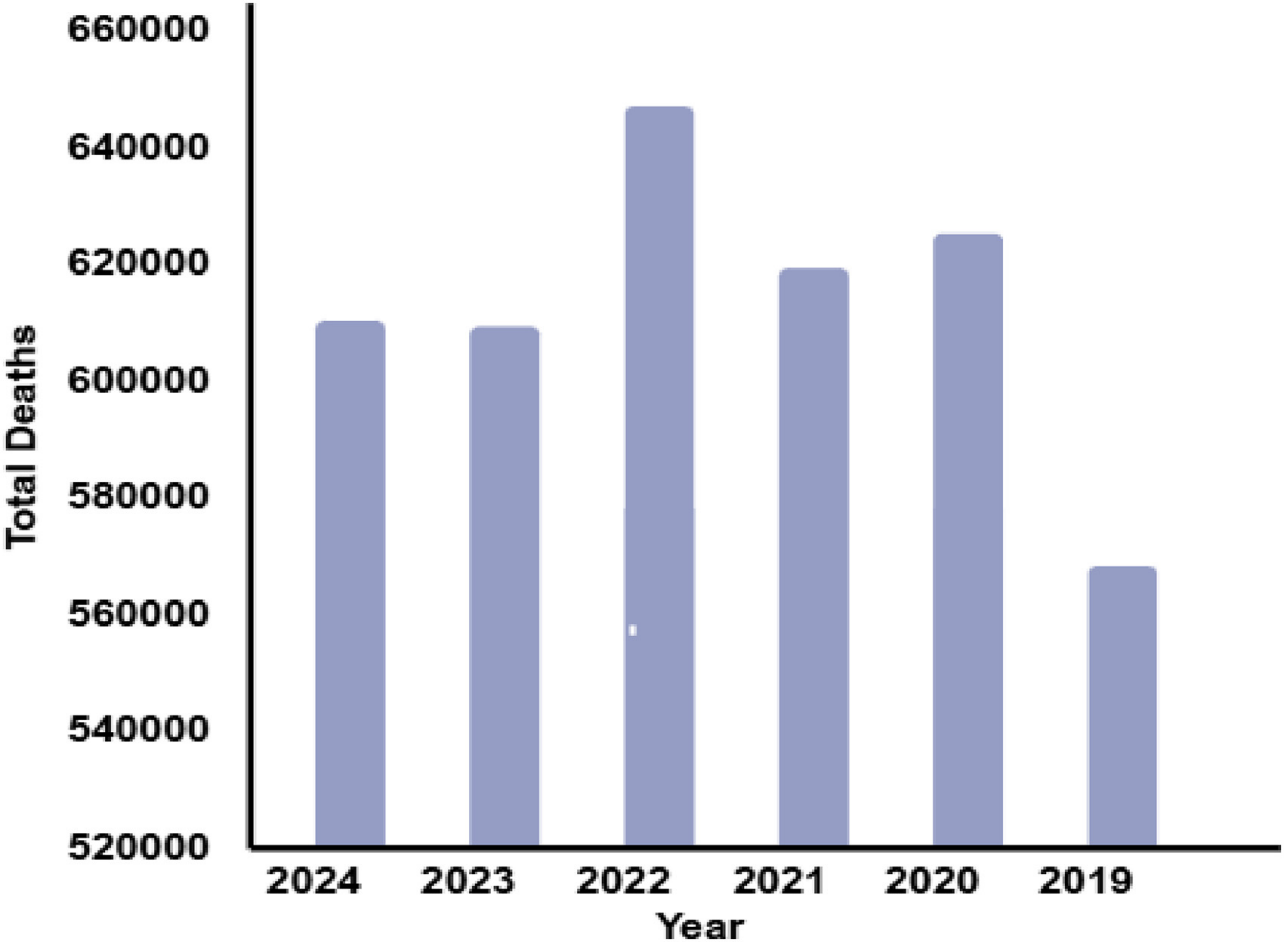
Total deaths recorded during 2019–2024.

**FIGURE 2:**
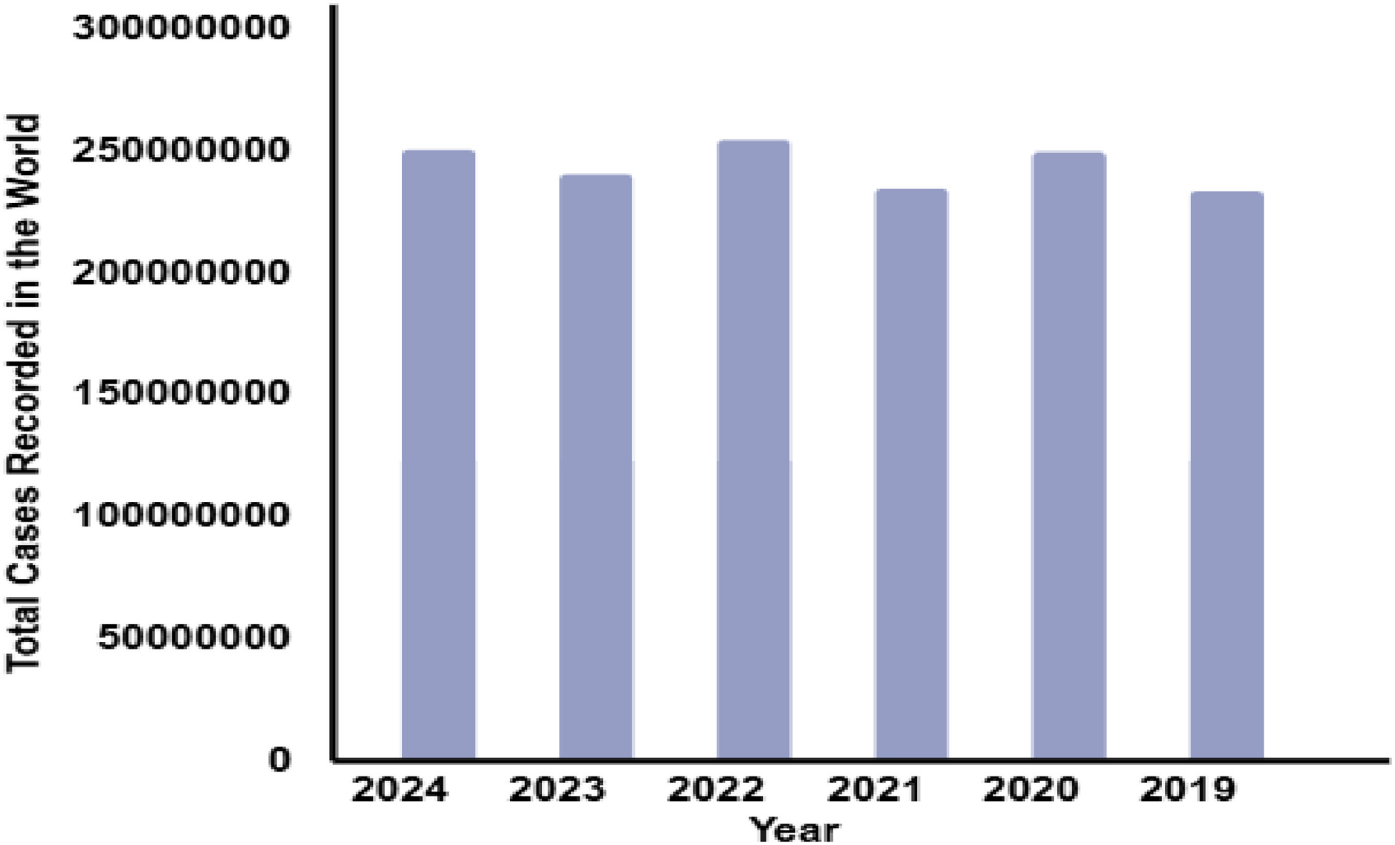
Total no. of cases recorded around the globe during 2019–2024.

Malaria, a debilitating disease caused by *Plasmodium* parasites, is a persistent global health challenge transmitted through the bites of infected female *Anopheles* mosquitoes. Despite decades of control efforts, malaria continues to impose a severe burden, with an estimated 229 million cases and 409,000 deaths reported in 2022 alone, disproportionately affecting low-resource regions, particularly in sub-Saharan Africa, which accounts for 92% of the cases and fatalities. Alarmingly, in 2023, the World Health Organization (WHO) recorded 214 million infections and 240,000 deaths, highlighting the pressing need for innovative and scalable solutions to tackle this disease effectively (1). Figures 1, 2, along with Table 1, depict the devastating impact of malaria globally between 2019 and 2024.

**TABLE 1:** Recorded cases of malaria around the globe.

S. No.	Year	Total cases recorded in the world	Total deaths
1.	2024	250 million	610,000
2.	2023	240 million	609,000
3.	2022	254 million	646,700
4.	2021	234 million	619,000
5.	2020	249 million	625,000
6.	2019	233 million	568,000

A novel ensemble framework that combines hard voting and adaptive weighted averaging, which distinguishes it from other CNN-based malaria detection techniques that typically employ fixed-weight averaging or simple majority voting. Stronger models are given more influence by adaptive weighted averaging, which dynamically allocates weights based on each model’s validation results, while hard voting ensures consensus reliability. While data augmentation, transfer learning, and CNN architectures are all popular techniques in medical imaging, this study is unique in that it combines numerous pre-trained models (VGG16, VGG19, DenseNet201, and ResNet50V2) with a custom CNN using an adaptive ensemble strategy. Unlike earlier malaria detection methods, which rely on a single model, this framework leverages the complimentary characteristics of numerous architectures, resulting in higher diagnostic accuracy and robustness. This integrated architecture distinguishes the proposed approach from previous methods while emphasizing its potential therapeutic usefulness. A key contribution of our study is the two-tiered ensemble technique, which enhances accuracy and resilience. Traditional diagnostic methods, such as the microscopic examination of blood smears, are labor-intensive, time-consuming, and prone to human error. These challenges are particularly pronounced in resource-constrained settings where the disease burden is highest (2). Modern advancements in machine learning (ML) have shown transformative potential in automating the diagnostic process, offering high accuracy and speed. Early studies have demonstrated the effectiveness of algorithms like Naive Bayes, Logistic Regression, Decision Tree, Support Vector Machine, and Random Forest in malaria detection tasks (3, 4). However, individual models often face limitations in generalizing across diverse datasets and addressing the complexities of malaria diagnosis. Ensemble learning, which combines predictions from multiple models, has emerged as a promising solution by leveraging the strengths of individual models while mitigating their weaknesses (5, 6). This approach enhances the robustness, precision, and reliability of automated diagnostic systems. Rural clinics and community health initiatives frequently encounter limited laboratory equipment, a scarcity of experienced microscopists, and delays in obtaining valid data. The proposed ensemble learning framework solves these issues by providing an automated, scalable method for accurate malaria diagnosis in low-resource environments. This strategy improves point-of-care screening and enables prompt treatments in underserved areas.

Compared to individual models, ensemble learning can improve robustness and generalization, which is why it was used in this work. The inherent diversity in medical imaging datasets may be difficult for standalone deep learning systems to properly capture and they are susceptible to overfitting. By utilizing the complementing qualities of several models, ensemble approaches reduce variation and increase stability in order to overcome these problems. Compared to single-model baselines, the suggested framework delivers higher diagnostic accuracy and reliability by combining VGG16, VGG19, DenseNet201, and a bespoke CNN.

Despite the widespread use of methods like data augmentation, transfer learning, and CNN-based architectures in medical imaging, this work is novel in that it uses an adaptive ensemble strategy to integrate several pre-trained models (VGG16, VGG19, DenseNet201, and ResNet50V2) with a custom CNN. Through the utilization of these architectures’ complimentary qualities, the suggested framework successfully lessens the drawbacks of each model alone. This demonstrates how unique our ensemble approach is and sets it apart from earlier malaria detection research.

Preprocessing plays a pivotal role in improving the quality of blood smear images, ensuring more accurate downstream processing steps such as feature extraction, cell segmentation, and classification (7, 8). For instance, applying smoothing filters like Gaussian and median filters effectively reduces noise and artifacts in microscopic images (9, 10). Previous research has explored the use of deep learning and machine learning techniques to enhance malaria diagnostic accuracy and efficiency (11, 12). Notably, methods focusing on *Plasmodium* detection in optical microscopy images have demonstrated significant advancements (13, 14). Smartphone-based deep learning tools have also shown potential in creating complex systems for real-time malaria detection (15–17), while innovative approaches using quantitative phase imaging or mid-infrared spectroscopy offer alternative diagnostic pathways (18, 19).

Efforts to combat malaria have predominantly targeted Plasmodium falciparum, the deadliest parasite species. However, global objectives, such as reducing malaria-related deaths and case incidence by 90% by 2030 and achieving complete eradication by 2040, necessitate a broader focus and innovative strategies. Emerging techniques like Generative Adversarial Networks (GANs) for image synthesis and denoising, or reverse convolution for image compression, further illustrate the potential of AI-driven solutions in addressing diagnostic challenges (20, 21). EfficientNet, a deep learning-based technique for identifying Malaria, is proposed using red blood cell pictures (22). A review using the PRISMA framework examines 50 studies (2015–2023) on AI-based malaria diagnosis, identifying common methodologies, important challenges such as inadequate validation and species categorization, and providing suggestions to improve future automated diagnostic and treatment efforts (23). Table 2 provides a comparative analysis of existing approaches for malaria prediction and diagnosis, highlighting their advantages and limitations.

**TABLE 2:** Comparative analysis of existing approaches for malaria predictions.

References	Approach	Technique	Performance	Limitations of the studies	Advantage of proposed model
Nayef et al. (24)	The proposed method analyses malaria-infected blood smear images using fuzzy logic and edge detection techniques. It uses mobile hardware for real-time analysis in remote settings.	The technique uses fuzzy logic-inspired edge detection, designing membership functions, fuzzy inference rules, defuzzification, and hardware optimization for real-time processing.	Efficient real-time edge detection for low-contrast images; performs well in remote settings.	Limited to edge detection and lacks deep learning’s advanced feature extraction capabilities; struggles with highly complex datasets.	Integrates deep learning and ensemble learning for robust feature extraction, achieving higher accuracy and generalization in complex datasets.
kaggle et al. (25)	Artificial intelligence (AI) tools for diagnosing microbiological diseases using machine learning and deep learning.	Uses supervised, unsupervised, and deep learning methods for genomics and bioinformatics to detect and predict diseases.	Highly accurate detection and prediction of microorganisms; supports pathologists.	Limited focus on malaria detection; not optimized for microscopic image analysis.	Tailored specifically for malaria detection with specialized CNN architectures and ensemble learning for precise classification of infected cells.
Bias et al. (26)	Automated system for detecting malaria-infected cells using Importance-Aware Balanced Group SoftMax (IBGS).	Employs CNNs with importance-aware weighting and balanced group SoftMax for minority class detection.	Improved recall for infected cells with reduced false negatives.	Limited to addressing class imbalance; may not generalize well across diverse datasets or variable image quality.	Utilizes data augmentation and ensemble learning to improve generalizability and resilience to image quality variations.
Ikerionwu et al. (12)	Employs sophisticated image analysis techniques to develop novel malaria diagnostic tools.	Focuses on automation for scalability and efficiency, reducing diagnosis time.	Success rates for models: SVM (27.9%), KNN (11.6%), Random Forest (7.0%).	Low performance metrics highlight inefficiency in real-world diagnostic scenarios.	Achieves state-of-the-art testing accuracy (97.93%) by integrating CNN and ensemble learning strategies.
Marletta et al. (27)	Weakly supervised deep learning strategy for malaria and sickle cell detection using Multiple Instance Learning (MIL).	CNNs with MIL framework predict sick cells using weak supervision and augmentation.	Competitive performance with reduced false negatives; scalable for clinical use.	Weak supervision may limit the model’s precision on highly detailed or complex datasets.	Fully supervised training and hyperparameter tuning ensure precise and robust performance across varied datasets.
Harvey et al. (13)	Machine learning and deep learning for precision malaria diagnosis via optical microscopy.	Forecasts malaria outbreaks using historical epidemic data with machine learning.	Achieves 30% accuracy and 99% recall for epidemic warnings.	Focuses on forecasting rather than direct cell-based malaria detection; lower accuracy for predictions.	Directly targets malaria detection at the cellular level, offering superior accuracy and recall.
Masud et al. (15)	Employs cutting-edge neural network architectures for accurate malaria parasite identification.	Uses advanced deep learning algorithms for healthcare and mobile-based diagnostics.	Achieves 97.30% accuracy for cell classification.	Limited ensemble strategies; lacks optimization for image quality inconsistencies.	Incorporates ensemble methods with adaptive learning strategies to enhance classification and reduce inconsistencies.
Fuhad et al. (16)	Deep learning techniques for automating malaria parasite detection in blood smears.	Combines CNN-based feature extraction with SVM/KNN classifiers, data augmentation, and auto-encoder optimization.	Achieves 99.23% accuracy with efficient operations.	Focuses on single deep learning models, which may overfit or lack robustness for diverse datasets.	Combines multiple deep learning approaches through ensemble learning for improved robustness and reduced overfitting.
Hemachandran et al. (10)	Framework for diagnosing malaria using CNN, MobileNetV2, and ResNet50.	Optimizes memory and computation for mobile deployment with compact CNN models.	ResNet50 achieves 97% accuracy and recall; f1-scores of 0.97 for both ResNet50 and MobileNetV2.	Mobile deployment prioritizes resource optimization over diagnostic accuracy.	Focuses on high diagnostic accuracy with ensemble learning while remaining adaptable for mobile applications.
Mwanga et al. (18)	Combines logistic regression and mid-infrared spectroscopy for malaria detection from dried blood spots.	Uses spectroscopy and logistic regression for field-friendly malaria detection.	Achieves 92% accuracy for P. falciparum detection; 85% for mixed infections.	Limited to specific data types (dried blood spots); lower accuracy for mixed infections.	Specializes in microscopic blood smear analysis, providing higher accuracy and versatility for varied infection types.
Sahu et al. (21)	Machine learning for predicting malaria risk from clinical data.	Logistic regression, decision tree, Gaussian NB, and random forest models; feature selection for prediction optimization.	Decision tree achieves highest accuracy (96.44%); random forest achieves 95.96% accuracy.	Relies on clinical data rather than image-based diagnosis; limited to prediction, not detection.	Excels in image-based malaria detection, providing direct diagnostic capabilities rather than risk prediction.
Sukumarran et al. (23)	A systematic evaluation of 50 studies (2015-2023) on ML/DL for malaria detection using blood smears.	Classical ML (SVM, KNN, XGBoost) and deep learning models (VGG, ResNet, DenseNet, MobileNet, EfficientNet, YOLO).	Deep learning, particularly transfer learning, outperforms machine learning, with accuracies frequently exceeding 95%.	The paper focused on binary classification, lacked cross-dataset validation, and overlooked multi-stage or species identification.	Lightweight transfer learning models integrate high accuracy and field efficiency.
Mujahid et al. (22)	Hybrid CNN with feature selection	CNN feature extraction + PSO for feature selection	Accuracy > 97% on NIH dataset	Single dataset, added complexity, limited interpretability	Reduces overfitting via feature selection

Despite significant advancements in malaria diagnosis using ML and deep learning (DL), existing approaches exhibit several limitations. Many studies rely on single-model architectures that struggle to generalize across diverse datasets, often leading to inconsistencies in performance when applied to real-world scenarios with varying image quality, noise levels, and clinical conditions (24, 28). Traditional diagnostic workflows, although enhanced with ML, frequently lack robustness in handling complex cases such as varying malaria severity, relapse patterns, and geographic differences in parasite strains (25, 26). Moreover, standalone models are prone to overfitting and may fail to adapt to the evolving nature of data, especially in resource-limited settings where input quality is inconsistent (27). Certain studies employ handcrafted feature extraction, which may inadvertently ignore critical patterns, while others focus solely on individual performance metrics without evaluating the practical application or scalability of the model. Recent developments in medical imaging have increasingly relied on machine learning and deep learning approaches to automate diagnosis. Multimodal masked autoencoders with adaptive masking have been used to accurately classify vitiligo stages, highlighting the power of deep learning for skin lesion analysis (29). Feature fusion techniques have been used to predict protein subcellular localization, demonstrating the value of combining various data views for better biological interpretation (30). Neuromorphic-enabled video-activated cell sorting is an example of AI-driven automation in cellular analysis (31, while deep neural networks have been used to predict dementia from imaging datasets (32, 34). Furthermore, bio-imaging-based machine learning algorithms have demonstrated great accuracy in breast cancer detection, highlighting the growing importance of computational techniques in assisting clinical decision-making (33). Reports of autoimmune consequences include hemophagocytic lymphohistiocytosis after encephalitis, while SIRT6 has been demonstrated to guard against inflammation in pulmonary endothelial cells caused by lipopolysaccharide.

By combining the complimentary advantages of several models, ensemble learning lowers overfitting and enhances generalization over a range of visual attributes. Compared to employing a single model, mixing multiple architectures improves diagnostic resilience and accuracy in malaria detection, where cell morphology and picture quality can vary greatly. In addition to accuracy, ensemble approaches can manage a greater variety of imaging settings, including changes in staining or lighting, which can mask important cellular properties. Ensembles reduce the biases of individual architectures by combining predictions via voting or weighted average, offering a more thorough evaluation of cells afflicted with malaria. Because pre-trained weights from sizable picture datasets offer a solid foundation—a benefit in medical imaging, where labeled data is frequently scarce and expensive—incorporating transfer learning significantly speeds up training. Performance can be further enhanced by ongoing observation and adjustment on fresh datasets. All things considered, the ensemble approach not only produces instant improvements in predicted accuracy but also creates a foundation for an adaptable system that can change in tandem with advancements in diagnostic techniques and imaging technology.

This paper addresses these gaps by proposing a novel ensemble learning framework that combines the strengths of multiple state-of-the-art transfer learning models, including VGG16, ResNet50V2, DenseNet201, and Custom Convolutional Neural Network (CNN). By aggregating predictions using advanced ensemble techniques such as weighted mean and hard voting, the proposed framework enhances diagnostic accuracy and robustness. Additionally, the systematic workflow introduced in this paper—encompassing image preprocessing, segmentation, feature selection, and classification—ensures a comprehensive and adaptable diagnostic pipeline. Unlike existing approaches, the proposed work prioritizes scalability and real-world applicability, demonstrating superior performance in diverse and challenging diagnostic scenarios. By leveraging ensemble learning, this work mitigates the limitations of individual models, providing a reliable and efficient solution to the complex challenges of malaria detection. The contributions of the proposed work include:

- A systematic four-stage diagnostic workflow (image pre-processing, segmentation, feature selection, and classification),
- Innovative ensemble techniques (weighted mean and hard voting),
- A comprehensive evaluation of the models, and
- Demonstrating its potential to address clinical challenges such as varying severity, relapse patterns, and geographic prevalence of malaria.

The proposed ensemble outperforms individual models, achieving enhanced accuracy and reliability, addressing the clinical complexities of malaria, including varying symptom severity and geographic prevalence. By bridging the gap between traditional diagnostic approaches and modern AI-driven techniques, this work aims to provide a scalable and robust solution for automated malaria detection. The findings hold significant implications for improving diagnostic capabilities, particularly in underserved regions, contributing to global malaria control and eradication efforts.

This method uses a weighted ensemble strategy designed for malaria cell image classification, in contrast to other ensemble-based systems that often use uniform averaging or simple majority voting. Through the integration of complimentary deep learning models (VGG16, DenseNet201, and VGG19), the suggested approach improves robustness and performance by utilizing each model’s distinct feature extraction capabilities.

The remainder of this paper is organized as follows: materials and methodologies are detailed, results and discussions are presented, and conclusions are drawn to outline the impact and future potential of this research.

## 2 Materials and methods

**FIGURE 3:**
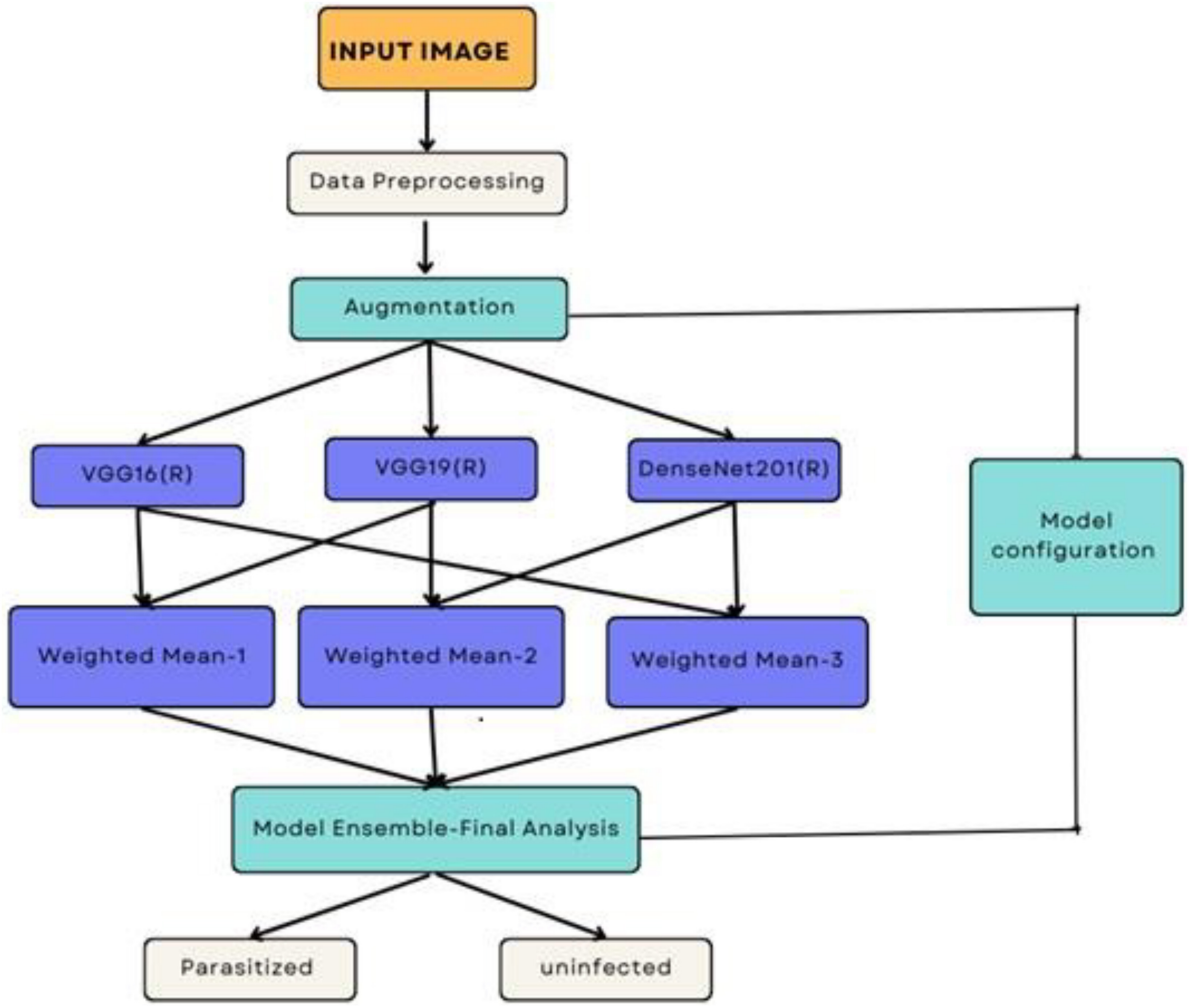
Process of performing the prediction.

This section outlines the dataset, preprocessing techniques, data augmentation, transfer learning frameworks, ensemble learning techniques, and custom CNN architecture employed in this paper to develop an efficient malaria detection system. Detailed steps, equations, and hyperparameter optimization are provided to ensure reproducibility. Figure 3 presents the process of performing the malaria prediction.

### 2.1 Dataset description

This research work utilizes a robust dataset sourced from the National Institutes of Health (NIH), comprising a total of 21,322 microscopic cell images, 13,779 of which were parasitized and 7,543 of which were not. The pictures were taken from thin Giemsa-stained blood smears. The analysis was conducted using only the NIH dataset; no other datasets were included. The restricted diversity of this dataset may limit the findings’ generalizability, despite the fact that it is frequently used in studies on malaria detection. The dataset plays a pivotal role in developing an ensemble learning-based system for malaria diagnosis. These images, captured at high resolution, are annotated to indicate whether the red blood cells are parasitized (infected with malaria) or uninfected. The characteristics of the dataset and the preprocessing strategies ensure consistency and accuracy for model training and evaluation. The dataset consists of:

- 13,779 parasitized cell images: Red blood cells infected with malaria parasites.
- 7,543 uninfected cell images: Healthy red blood cells devoid of infection.

**FIGURE 4:**
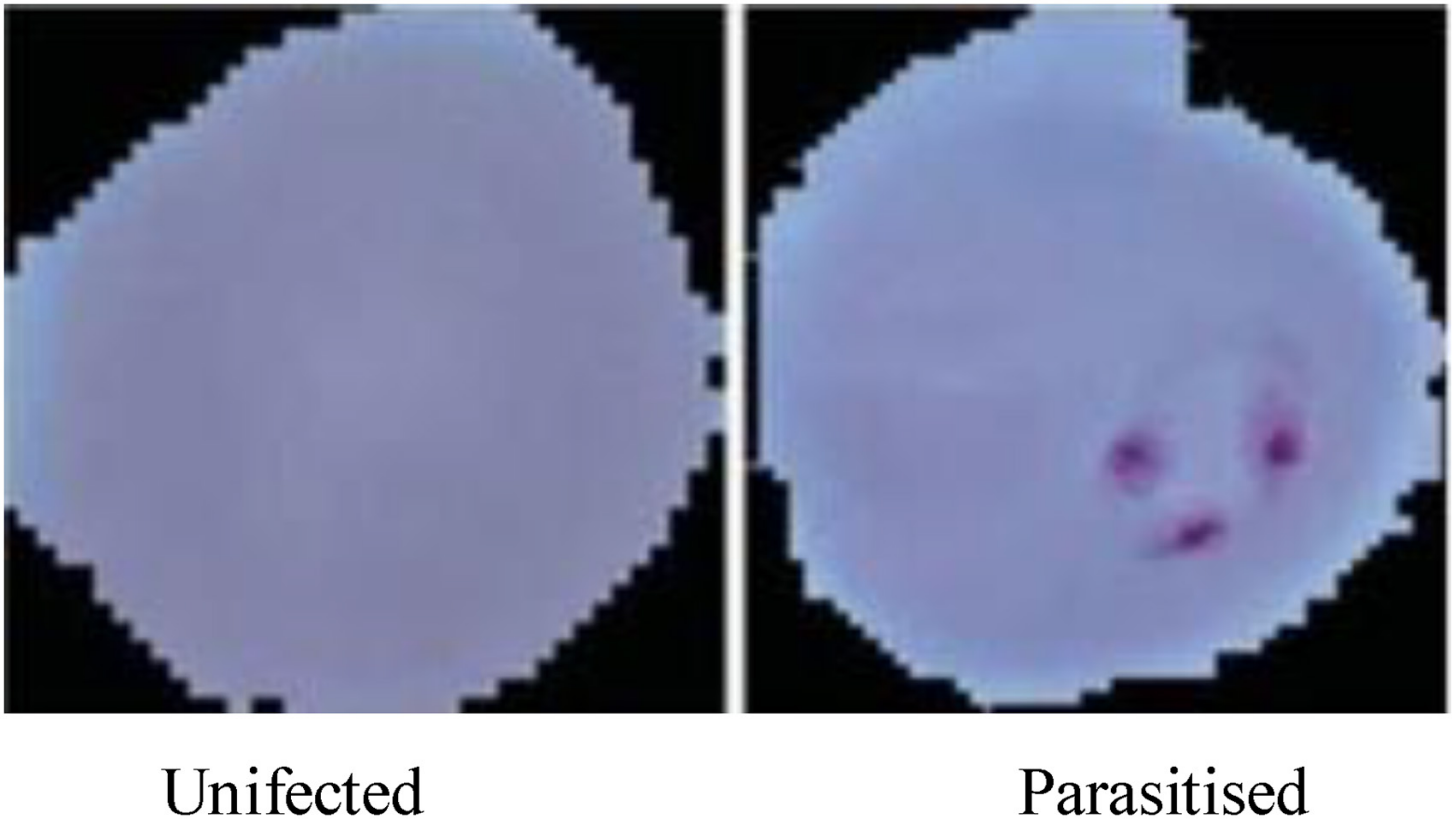
Microscopic images of malaria used for malaria prediction.

**FIGURE 5:**
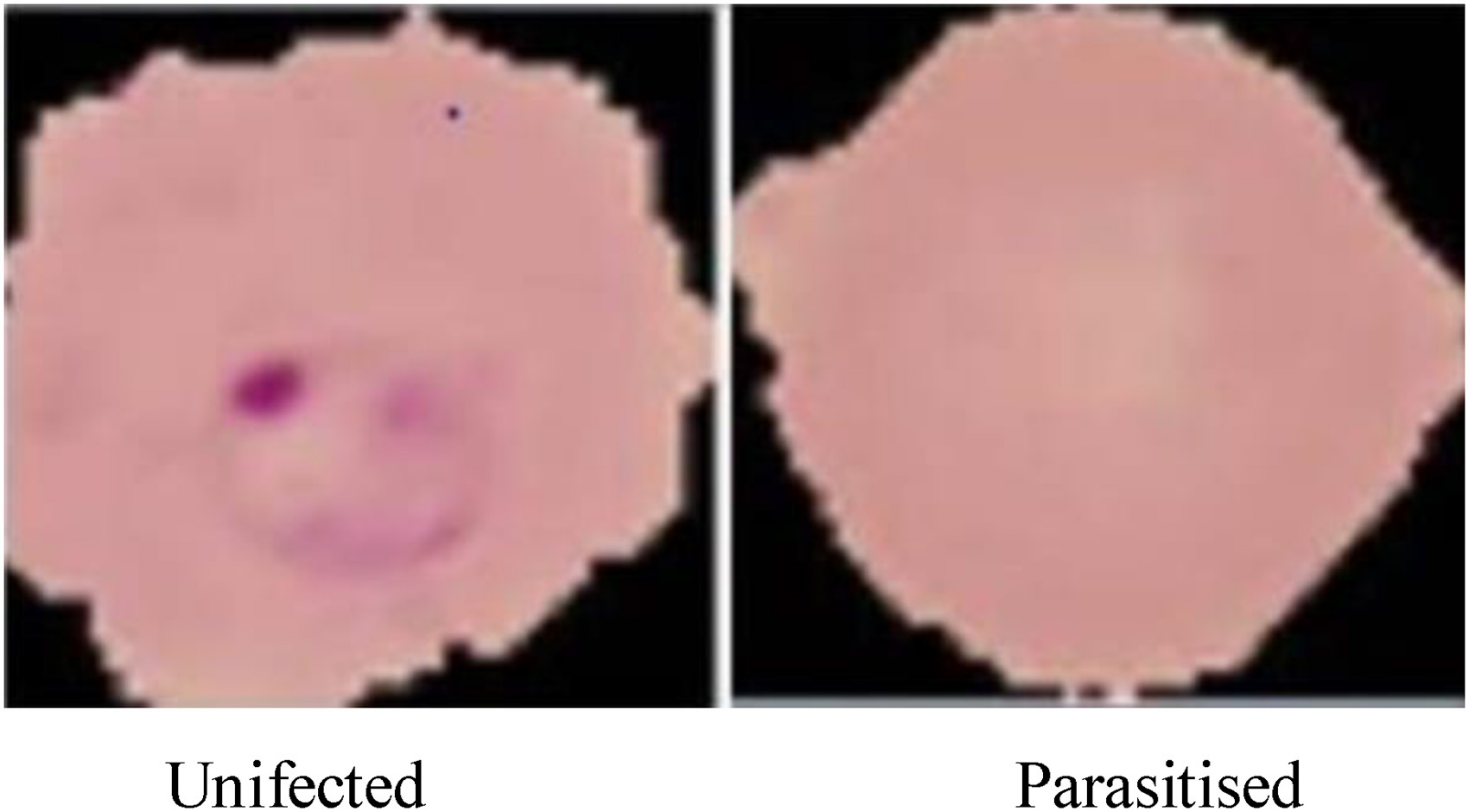
Resized pictures of malaria cells used for malaria prediction.

The dataset is visually rich, capturing a diverse range of cells across different conditions and visual features, as shown in Figures 4, 5. Each image in the dataset is labeled, aiding in supervised learning for binary classification. Despite its overall quality, a subset of the dataset revealed mislabelling issues, impacting approximately 5% of entries:

- 750 uninfected cells were mislabelled as parasitized.
- 647 parasitized cells were mislabelled as uninfected.

These errors were identified and rectified with the help of domain experts to ensure that the training data was accurate and reliable.

The National Institutes of Health (NIH) made 21,322 cell photos available on Kaggle, which served as the dataset for this research. It is made up of 13,780 parasitised and 7,543 uninfected cells that were taken from thin blood smear slides stained with Giemsa. To ensure reader clarity, this is specifically acknowledging both the NIH and Kaggle sources in the Methods section rather than referring to the dataset alone in the supplemental materials. The dataset exhibits the following properties that necessitate specific preprocessing steps for effective use in machine learning:

- Image dimensions:
- Original image sizes range between 110 and 150 pixels.
- To ensure uniformity and compatibility with the CNN architecture, images were resized to 64 × 64 pixels. This reduction maintains essential features while optimizing computational efficiency.
- For train-test split, the dataset was divided into:
- 80% for training: 17,057 images used for model training and validation, enabling the network to learn from a diverse set of features.
- 20% for testing: 4,265 images reserved for evaluating the generalization and robustness of the trained model.
- Visual representation:
- Figure 4, displays examples of parasitized and uninfected cells, illustrating the diversity and complexity of the dataset.
- Figure 5, demonstrates the resized images post-preprocessing, showcasing uniform dimensions critical for training deep learning models.

This NIH dataset serves as an invaluable resource for malaria prediction due to its scale, diversity, and richness. The challenges posed by mislabelled entries indicate the importance of data quality and domain expertise in ensuring accurate model training. Furthermore, the careful splitting of the dataset ensures a balance between training efficiency and testing reliability, forming the foundation for robust model evaluation. By systematically preparing and preprocessing this dataset, the paper establishes a solid groundwork for building an advanced machine-learning system capable of diagnosing malaria with high precision and reliability.

### 2.2 Data preprocessing

Effective preprocessing is a critical step in any machine learning workflow, particularly in medical image analysis. For this study, a systematic data preprocessing pipeline was implemented to ensure high-quality, standardized inputs for model training and evaluation. By normalizing image data, reducing noise, and addressing class imbalances, the preprocessing stage lays a strong foundation for developing an accurate and robust malaria diagnosis system.

#### 2.2.1 Normalization

Normalization is essential to scale image pixel values to a uniform range, thereby enhancing computational efficiency and model convergence during training. For this dataset, pixel values ranging from 0 to 255 were scaled to a normalized range of (0,1). The raw pixel values are scaled by Equation 1 from [0,255] to [0,1], guaranteeing that all inputs are normalized before to being supplied to the network (1).


$$I_{n\,o\,r\,m\,a\,l\,i\,z\,e\,d}=\frac{I_{o\,r\,i\,g\,i\,n\,a\,l}}{255}$$ (1)

Here:

- *I*_*original*_ represents the original pixel intensity values.
- *I*_*normalized*_ refers to the scaled intensity values.

This step ensures that all input features are on a similar scale, preventing certain pixels from disproportionately influencing the model. Additionally, it facilitates faster and more stable learning by neural networks.

#### 2.2.2 Noise reduction

Medical images often contain visual artifacts and noise, which can obscure critical features necessary for accurate classification. To mitigate these issues:

- **Gaussian filters:** Applied to blur the images and reduce high-frequency noise while preserving edges. This technique ensures smooth transitions between pixel intensities, enhancing feature extraction.
- **Median filters:** Used to remove salt-and-pepper noise, replacing each pixel’s value with the median of its neighborhood. This operation effectively preserves edges while eliminating outliers.

By reducing noise, these filtering methods improve the clarity of microscopic cell images, ensuring that the machine learning model focuses on relevant diagnostic features rather than extraneous artifacts.

#### 2.2.3 Class balance

Class imbalance and labeling errors can significantly impact the training process, leading to biased or inaccurate models. Addressing these issues involved two critical steps:

- **Mislabelling corrections:** Experts in the hematology domain provided input to correct the dataset/s mislabeled samples. Two experts independently reviewed images that were thought to be possibly mislabeled, and labels were changed depending on their agreement. In order to guarantee label correctness and dependability before model training, any disputes were settled through dialogue.
- Expert validation was employed to rectify labeling errors in the dataset.
- Specifically, 750 uninfected cells initially mislabelled as parasitized and 647 parasitized cells mislabeled as uninfected were corrected, ensuring a reliable ground truth for training.
- **Data augmentation for class imbalance:**
- To counter class imbalance, various data augmentation techniques were employed. These included random rotations, flips, zooms, and scaling, as detailed in the augmentation pipeline.
- Augmentation not only increased the quantity of training data but also introduced variability, enabling the model to generalize better to unseen samples.
- **Mislabeled data correction:**

Experts in hematology assessed images with dubious classifications, and consensus was reached to make adjustments. A two-step procedure that involved independent evaluation by two experts and conversation to settle any discrepancies was used to guarantee reliability. This method generated precise, superior labels for training the model.

Hematology specialists examined every image that was marked as suspicious or possibly mislabeled in order to guarantee the validity of the data. Expert consensus led to revisions in the labels. To ensure trustworthiness, a two-step verification method was used:

- Independent evaluation by two experts.
- Settlement of any disputes to arrive at a final consensus.

By reducing errors that could impair model performance, this process guarantees that the dataset used for model training is precise and of high quality.

This preprocessing pipeline ensures that the dataset is clean, balanced, and normalized for optimal performance during training. The integration of normalization, noise reduction, and class balancing techniques addresses key challenges associated with medical image datasets, such as variability in image quality and class distribution. Consequently, these preprocessing strategies enhance the robustness and reliability of the ensemble learning-based malaria detection system.

### 2.3 Data augmentation

Data augmentation is a crucial technique in DL, particularly for medical imaging, where obtaining large and diverse datasets can be challenging. In this study, data augmentation techniques were employed to expand the dataset artificially, introducing variations that help improve the model’s ability to generalize across unseen samples. By applying a range of transformations, the augmented dataset retains its original diversity while simulating real-world variability in microscopic images. To enhance the dataset and improve model performance, several augmentation techniques were applied:

- Random rotation: Images were rotated randomly within a range of±30°. This introduces angular variability, helping the model learn features independent of orientation.
- Zooming: A random zoom transformation of up to 20% was applied. This simulates variations in magnification during image acquisition, improving the model’s robustness to changes in scale.
- Flipping: Horizontal and vertical flips were incorporated to ensure that the model recognizes malaria-infected cells regardless of their alignment or orientation.
- Rescaling: All pixel values were rescaled to the range (0,1) using a factor of 1/255, consistent with normalization practices, to ensure uniformity and compatibility with the model input pipeline.

**FIGURE 6:**
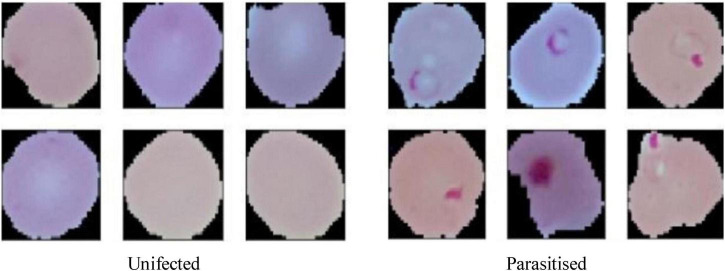
Augmented cells used for malaria prediction.

Table 3 outlines the parameter values for each augmentation technique applied in this paper, while Figure 6 provides visual examples of the transformed images. These settings were carefully chosen to preserve the biological relevance of the dataset while introducing meaningful variability. These transformations were implemented uniformly across all images in the dataset, ensuring consistency during preprocessing.

**TABLE 3:** Parameter values for each augmentation technique.

Augmentation technique	Parameter value
Random rotation	± *30*
Zoom	Up to 20%
Flipping	Horizontal and vertical
Rescaling	1/255

By introducing a wide range of transformations, the augmented dataset helps the model generalize better to unseen data, reducing the risk of overfitting. While the data augmentation effectively balances classes and increases sample diversity, addressing inherent limitations in the original dataset. Variations such as rotations, flips, and zooms prepare the model to handle real-world scenarios, where image orientation and magnification may vary. Figure 6 illustrates examples of augmented images, highlighting the diversity introduced by the applied transformations. These augmented images demonstrate how the dataset maintains its biological characteristics while incorporating meaningful variability, ensuring that the malaria detection model can perform accurately and robustly across diverse input scenarios. By employing these augmentation techniques, the paper ensures a rich and varied training dataset, which is pivotal for the success of the ensemble-based malaria prediction system.

### 2.4 Transfer learning frameworks

To leverage the power of existing state-of-the-art DL architectures, this paper utilized pre-trained models—VGG16, VGG19 (34), and DenseNet201—within a transfer learning framework. These models, pre-trained on large-scale image datasets like ImageNet, were fine-tuned to adapt to the specific task of malaria diagnosis using microscopic images. By modifying their architecture and hyperparameters, these models were optimized for accurate classification of parasitized and uninfected cells.

The pre-trained models were utilized for feature extraction, taking advantage of their hierarchical feature representation capabilities (35–38). These models are particularly effective in extracting both low-level and high-level features, which are crucial for identifying malaria-infected cells. The key adjustments made to adapt these models include, modification of dense layers; The final dense layers of the pre-trained models were replaced with fully connected layers tailored for binary classification (infected vs. uninfected). This adjustment ensures that the models focus on malaria-specific features, rather than the general-purpose features learned during pre-training. Several hyperparameters were optimized to enhance the performance of the models, the batch size was **s**et to 32, ensuring a balanced trade-off between memory efficiency and gradient update frequency. Epochs were defined as 100, allowing sufficient iterations for the model to learn from the training data, and the learning rate was adjusted to 10^4^, providing a stable and gradual learning process, avoiding overshooting or stagnation during optimization.

The strength of transfer learning lies in its ability to utilize pre-trained layers for hierarchical feature extraction. The initial layers of VGG16, VGG19, and DenseNet201 focus on capturing fundamental patterns like edges and textures, which are critical for distinguishing cellular structures in microscopic images. While the deeper layers are responsible for learning more abstract representations, such as the presence of parasitic artifacts, enabling precise classification. The fine-tuned models were trained and validated using the pre-processed dataset, ensuring that they adapt effectively to the malaria diagnosis task. Specific techniques employed to enhance optimization include, utilizing the Adam optimizer to dynamically adjusts learning rates for each parameter, and accelerate convergence while minimizing errors. The loss function was tailored to the binary nature of the classification task, effectively minimizing the gap between predicted and true labels. Also, regularization methods, such as L2 regularization and dropout layers, were incorporated to reduce overfitting and enhance the robustness of the models.

Overall, leveraging pre-trained models significantly reduces the time and computational resources required for training, as most of the feature extraction is already accomplished. The hierarchical feature extraction capabilities of VGG16, VGG19, and DenseNet201 improve the model’s ability to identify parasitized cells accurately. Fine-tuning these architectures ensures that the models generalize well to the specific dataset while retaining their high baseline performance. By incorporating transfer learning frameworks, this paper effectively harnesses the power of pre-trained DL models, significantly enhancing the accuracy and efficiency of malaria diagnosis. These models form the backbone of the proposed ensemble method, which further improves performance by combining their individual strengths. For transfer learning, we used VGG16, VGG19, DenseNet201, and ResNet50V2 as foundation models. These designs were chosen because of their computational efficiency, availability of pretrained weights, and demonstrated efficacy in medical imaging. The focus on testing a well-established set of CNN models to assure repeatability and increased computing needs prevented the inclusion of newer architectures, such as EfficientNet and Vision Transformers, despite their promising performance in recent studies. These more recent architectures may be investigated in future research for possible performance improvements.

### 2.5 Ensemble learning methodology

To achieve superior robustness and accuracy in malaria diagnosis, an ensemble learning methodology was adopted. This technique combines the predictions of multiple models, effectively harnessing their individual strengths to deliver enhanced diagnostic precision. The ensemble method employed two strategies: Weighted Mean Ensemble and Hard Voting.

The Weighted Mean Ensemble method calculates a weighted average of predictions from multiple models to produce the final output. The formula governing this approach is:


$$P_{e\,n\,s\,e\,m\,b\,l\,e}=\sum_{i=1}^{n}w_{i}\,P_{i}$$ (2)

Where:

- *P*_*ensemble*_: The final ensemble prediction.
- *w_i_*: The weight assigned to the i*^th^* model, reflecting its contribution.
- *P_i_*: The prediction probability from the i*^th^* model.
- *n*: The total number of models in the ensemble.

Equation 2 defines a weighted average of the individual model outputs, which provides the final ensemble prediction. Three model combinations were used, with specific weights assigned to each. Model 1 was a combination of VGG16 and VGG19 with weights, *w*_1_0.7, and *w*_2_0.6. This pairing utilizes the complementary feature extraction capabilities of VGG16 and VGG19. Model 2 was a combination of VGG19 and DenseNet201 where weights, *w*_1_0.6, and *w*_2_0.5. This model benefits from DenseNet201’s efficient feature reuse and VGG19’s deeper network structure. Model 3 was a combination of VGG16 and DenseNet201 were weights, *w*_1_0.4, and *w*_2_0.5. This combination balances the simplicity of VGG16 with the densely connected architecture of DenseNet201. These weighted combinations ensure that the strengths of each model are appropriately prioritized in the final prediction.

The Hard Voting approach determines the final prediction based on a majority vote among the individual model predictions. The mathematical representation is given in Equation 3:


$$P_{f\,i\,n\,a\,l}=m\,o\,d\,e\,\left(P_{1},P_{2},\ldots ,P_{n}\right)$$ (3)

Where:

- *P*_*final*_: The final aggregated prediction.
- *P*_1_,*P*_2_,…,*P*_*n*_: Predictions from individual models.

In this approach, each model contributes equally, and the prediction category with the majority votes is selected as the final output. Hard voting is particularly effective in scenarios where the models have varied prediction patterns, ensuring consensus-driven accuracy.

**FIGURE 7:**
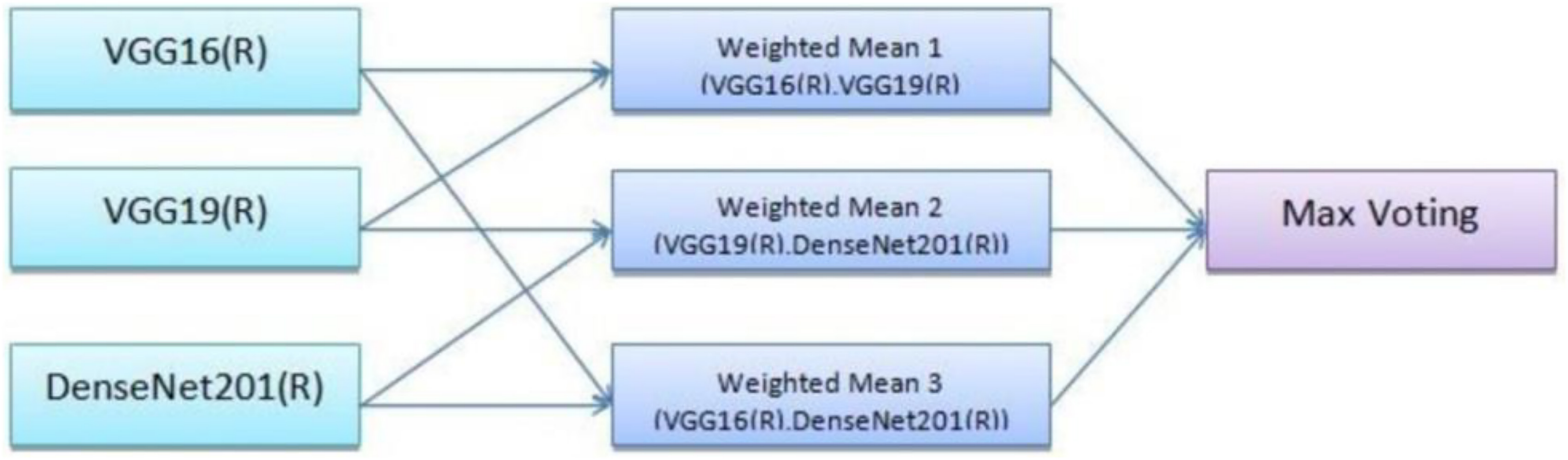
Working of the ensemble approach.

**FIGURE 8:**
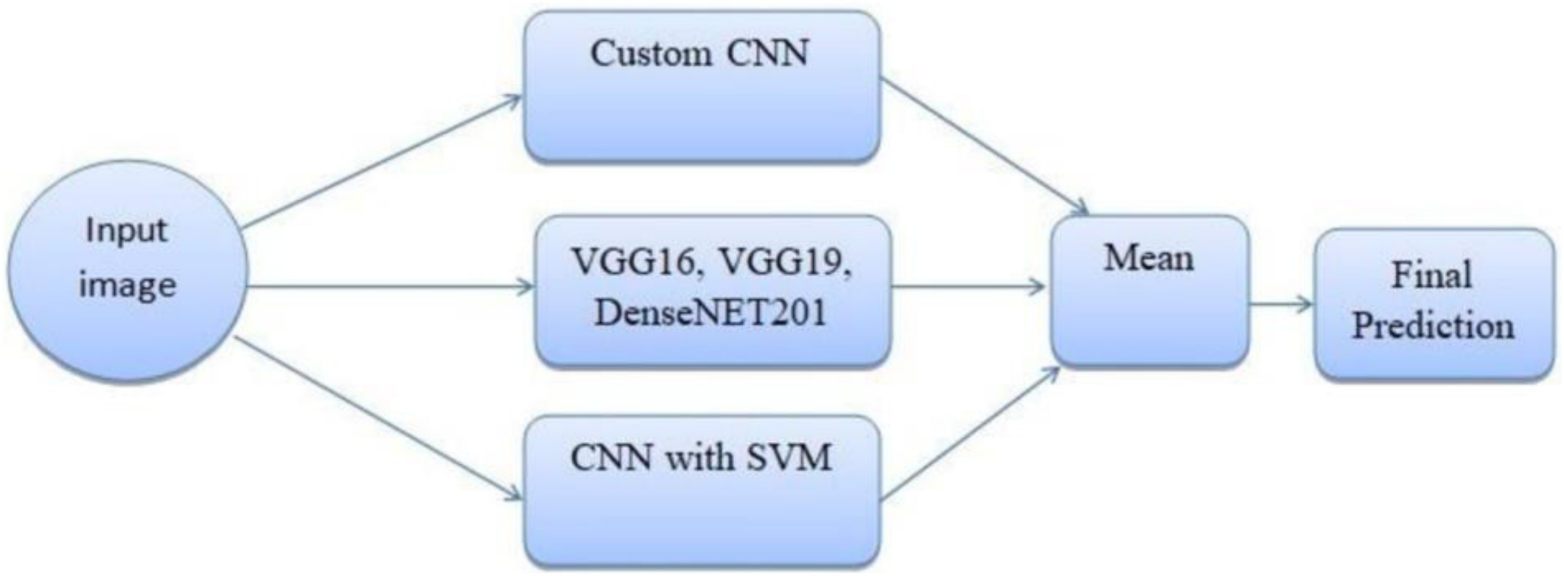
Ensemble method for malaria prediction.

**FIGURE 9:**
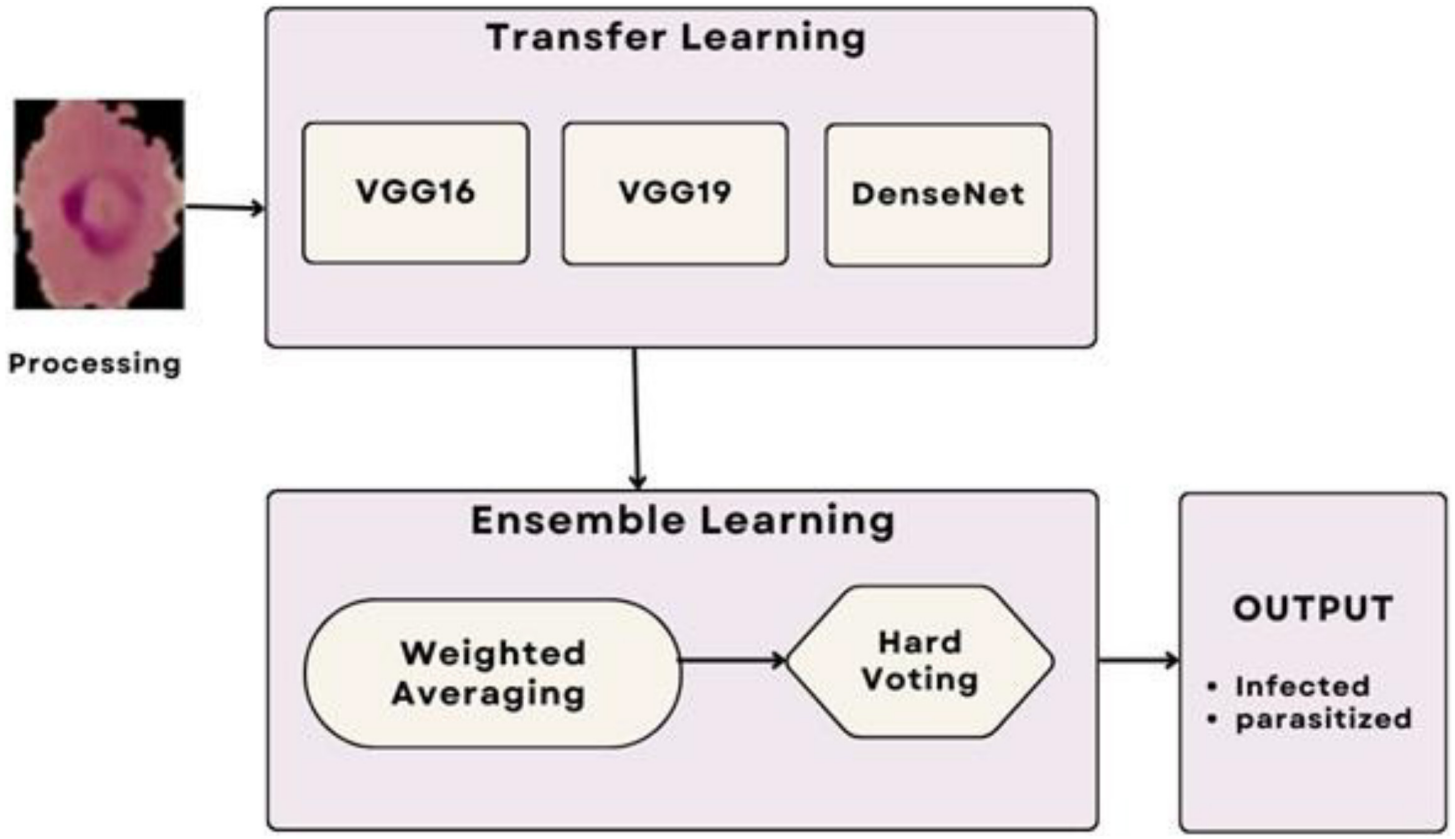
Workflow of the proposed ensemble learning for malaria parasite detection.

The ensemble methodology offers several advantages; by combining multiple models, the ensemble reduces the impact of errors from individual predictions. The ensemble approach mitigates the risks of overfitting that may occur when relying on a single model. The weighted mean approach allows fine-tuning of weights to adapt to specific datasets or classification challenges. Adaptive weighted averaging and hard voting are used in the ensemble to integrate base models. Adaptive weighting dynamically distributes weights to each model based on validation results, giving stronger models more sway than traditional fixed-weight averaging. The ensemble outperforms fixed-weight techniques in terms of accuracy and robustness thanks to this data-driven methodology. For example, compared to a fixed-weight ensemble, grid search on the validation set produced better overall validation accuracy by identifying the ideal weights for every model combination. Figure 7 illustrates the workflow of the ensemble learning methodology, showcasing the integration of predictions from multiple models through weighted averaging and hard voting mechanisms. Figure 8 visually represents the ensemble approach, highlighting the intricacies of this innovative methodology. Figure 9 illustrates the wfiguorkflow of the proposed ensemble learning for malaria parasite detection.Overall, this ensemble strategy forms a crucial part of the proposed malaria prediction framework, ensuring reliable and precise classification across diverse scenarios.

Three ensemble combinations were employed, and a certain weight was given to each model. With validation accuracy serving as the optimization criterion, the weights were found using a grid search in increments of 0.1 within the range (0.1–0.9). Grid search was chosen because it was straightforward, reproducible, and appropriate for computing restrictions, even though Bayesian optimization was taken into consideration. In the process, models that performed better on their own, such VGG19 and DenseNet201, were inherently given more weight. For instance, the ideal weights in Model 1 (VGG16+VGG19) were 0.4 and 0.6, respectively. Table 12 provides an overview of the ideal weights for every ensemble combination.

### 2.6 Algorithm: malaria detection ensemble framework

#### 2.6.1 Dataset D: microscopic cell images as input

Predicted class labels (infected or uninfected) are the output.

1. Preprocessing

- Normalize pixel values to fall between 0 and 1
- Use Gaussian and median filters to reduce noise
- Make all pictures 64 by 64 pixels.
- Use expert review to validate and correct samples that have been incorrectly labeled.

2. Augmenting data

Implement random rotations (± 15°), flip horizontally and vertically, apply random zoom up to 20%, and rescale to maintain consistent input dimensions.

3. Utilizing transfer learning for feature extraction

- Adjust DenseNet201, ResNet50V2, VGG16, and VGG19.
- Use a binary classification head in place of the last layers c. Run each model on the training subset.

4. Collective forecasting

- Compile each model’s Pi probability outputs.
- Make ensemble weights better Wi with grid search
- Determine the ultimate weighted score:

$$P_{e\,n\,s\,e\,m\,b\,l\,e}=\sum_{i=1}^{n}w_{i}\,P_{i}$$ (4)

- To break classification ties, use hard voting.

5. Personalized CNN Integration

Train the suggested CNN to extract more features; evaluate its performance against transfer learning models; and, if desired, incorporate CNN outputs into the ensemble.

6. Assessment of the Model

- Divide the dataset into 80% training and 20% testing.
- Use F1-score, Accuracy, Precision, and Recall to evaluate performance.
- Note the typical inference time for each picture.

7. Return: The ensemble’s final class label prediction

### 2.7 Custom CNN Architecture

To complement the transfer learning frameworks and ensemble methodologies, a proprietary CNN architecture was developed. This model was specifically designed to handle the unique challenges of malaria cell classification, such as feature variability and dataset imbalance. The architecture comprises several optimized components aimed at enhancing feature extraction, generalization, and classification accuracy. The custom CNN architecture developed for malaria cell classification is designed to effectively extract spatial features, mitigate overfitting, and improve generalization.

Convolutional layers form the backbone of the CNN and are responsible for extracting hierarchical spatial features from input images. These layers apply convolution operations using kernels to detect patterns such as edges, textures, and shapes. The first layer utilizes 32 filters with a 5 × 5 kernel size. Larger kernel size in the first layer helps in capturing more general features like shapes and contours. The second layer **a**lso comprises 32 filters but uses a smaller 3 × 3 kernel size to focus on finer details and local features. The Rectified Linear Unit (ReLU) is employed for non-linearity. ReLU prevents negative outputs and reduces the risk of vanishing gradient issues, accelerating convergence during training. It is given as:


R⁢(x)=m⁢a⁢x⁢(0,x) (5)

Batch normalization standardizes the outputs of each layer to reduce internal covariate shift, thereby stabilizing the learning process. It is defined as:


$$\text{μ }=\;\frac{1}{n}\sum_{i}N\left(i\right)$$ (6)

$$\text{σ }=\;\frac{1}{n}\sum_{i}\left(N_{\left(i\right)}\;-\;\mu \right)$$ (7)

$$N_{n\,o\,r\,m}^{\left(i\right)}=N\,\left(i\right)-\mu /\sqrt{}\,\sigma \,2+\varepsilon$$ (8)

Here:

- *N*^(*i*)^: Input to the normalization layer.
- μ: Mean of the inputs.
- σ^2^: Variance of the inputs.
- ε: A small constant to avoid division by zero.

This enhances training stability and allows the use of higher learning rates, speeding up the optimization process. The batch normalization process involves averaging activation values across the batch and determining standard deviation (Equations 4, 5). To achieve the normalization of the activation vector N[l], Equation 6 is utilized. Additionally, the layer output N(i) is computed by employing with trainable parameters γ and β, expressed in Equation 7. By centering the input activations to zero mean and scaling them to unit variance, Equation 8 normalizes them and ensures stable learning dynamics. By introducing trainable scale (γ) and shift (β) parameters, Equation 9 enables the network to regain representational flexibility following normalization.


$$N=\gamma *N_{n\,o\,r\,m}^{\left(i\right)}+\beta$$ (9)

Dropout is a regularization technique to combat overfitting, particularly in deep networks with large parameter spaces. During training, 20% of neurons are randomly deactivated which forces the model to learn more distributed and robust representations instead of relying heavily on specific neurons. It encourages the network to learn more robust representations by preventing reliance on specific neurons. Dense (fully connected) layers consolidate the features extracted by convolutional layers and produce predictions. Layer 1 contains 4096 neurons and applies L2 regularization to penalize large weights, further controlling overfitting. Layer 2 includes 1,024 neurons, refining the feature mappings generated in the previous layers. These layers integrate all the features extracted and prepare them for final classification. The SoftMax layer computes probabilities for each class, enabling binary classification of parasitized and uninfected cells. It produces normalized probabilities between 0 and 1, ensuring interpretability. It is given as:


$$s\,\left(z_{i}\right)=\frac{e_{i}^{z}}{\sum_{j=1}^{k}e_{j}^{z}}$$ (10)

Sparse categorical cross-entropy is utilized to measure the divergence between the true and predicted class probabilities. Although typically applied in multi-class scenarios, this loss function is particularly useful for binary tasks as it precisely captures classification errors. It is defined as:


$$L\,o\,s\,s=-\frac{1}{N}\,\sum_{i=1}^{N}y_{i}\,l\,o\,g$$ (11)

Where:

- *N*: Number of samples.
- *y_i_*: True label of the i-th sample.
- ${\hat{y}}_{i}$: Predicted probability for the true class.

**FIGURE 10:**
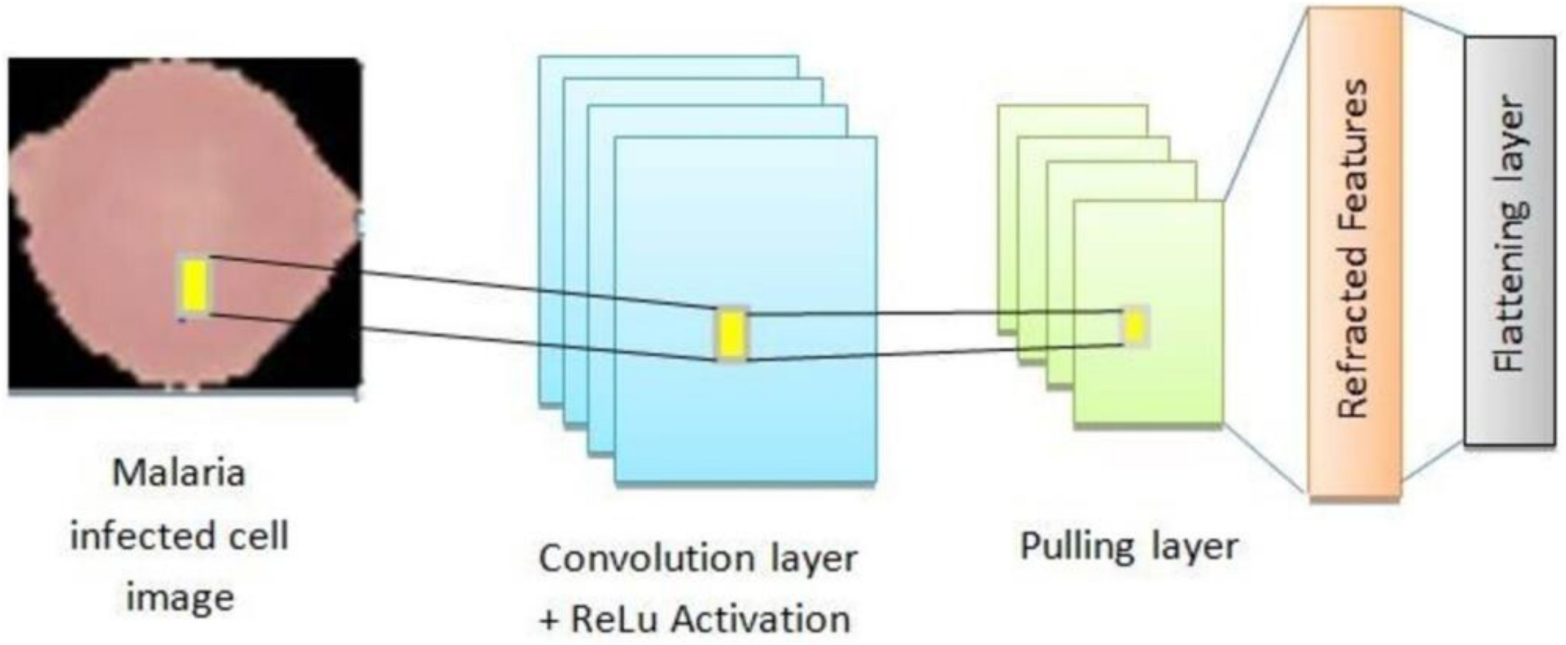
Architecture of custom CNN architecture for malaria prediction.

**FIGURE 11:**
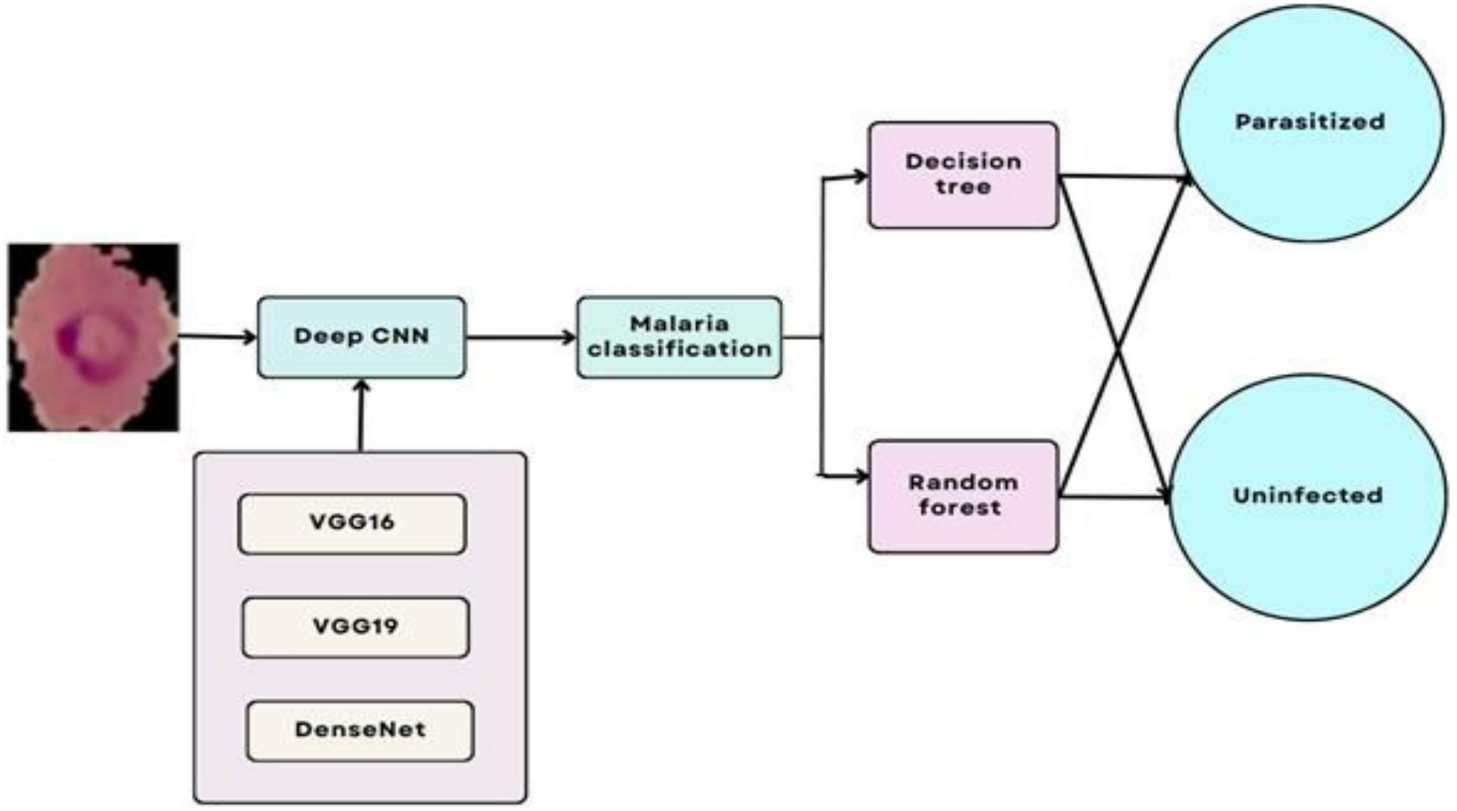
Architecture of CNN-ML classifier model for malaria prediction.

**FIGURE 12:**
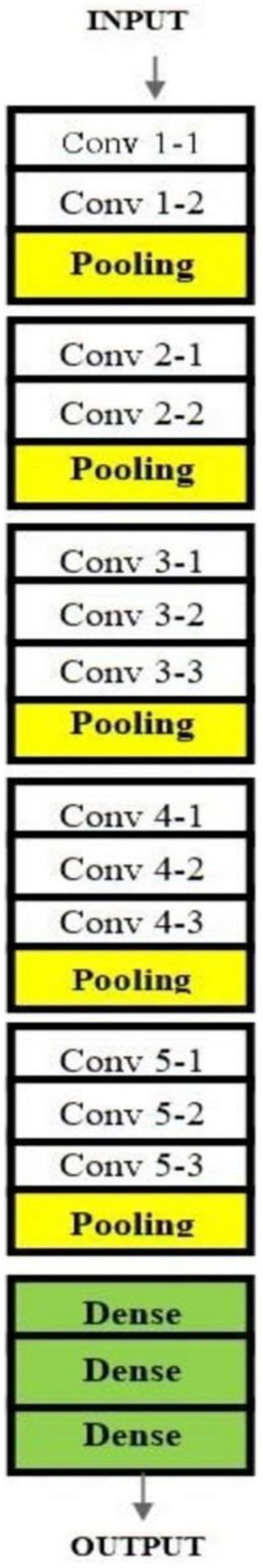
Architecture of VGG16 used for malaria prediction.

**FIGURE 13:**
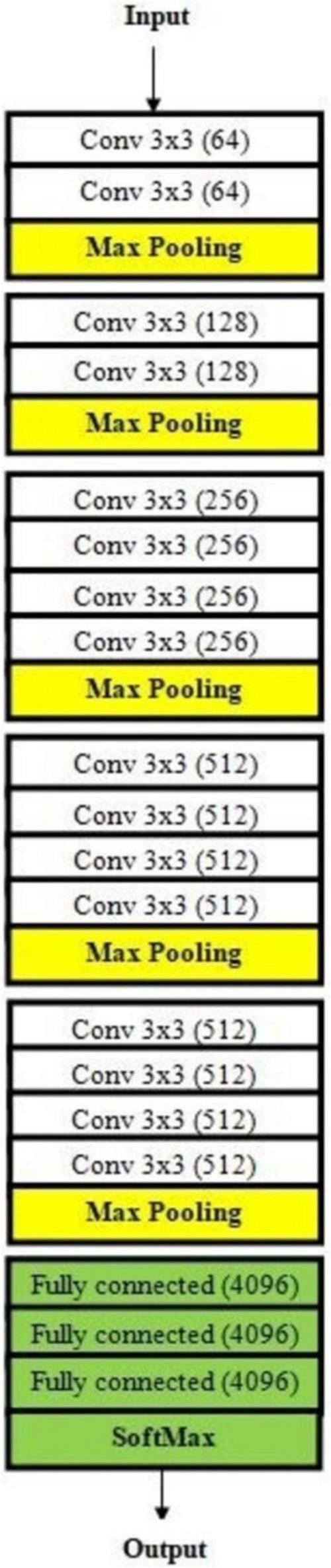
Architecture of VGG19 used for malaria prediction.

**FIGURE 14:**
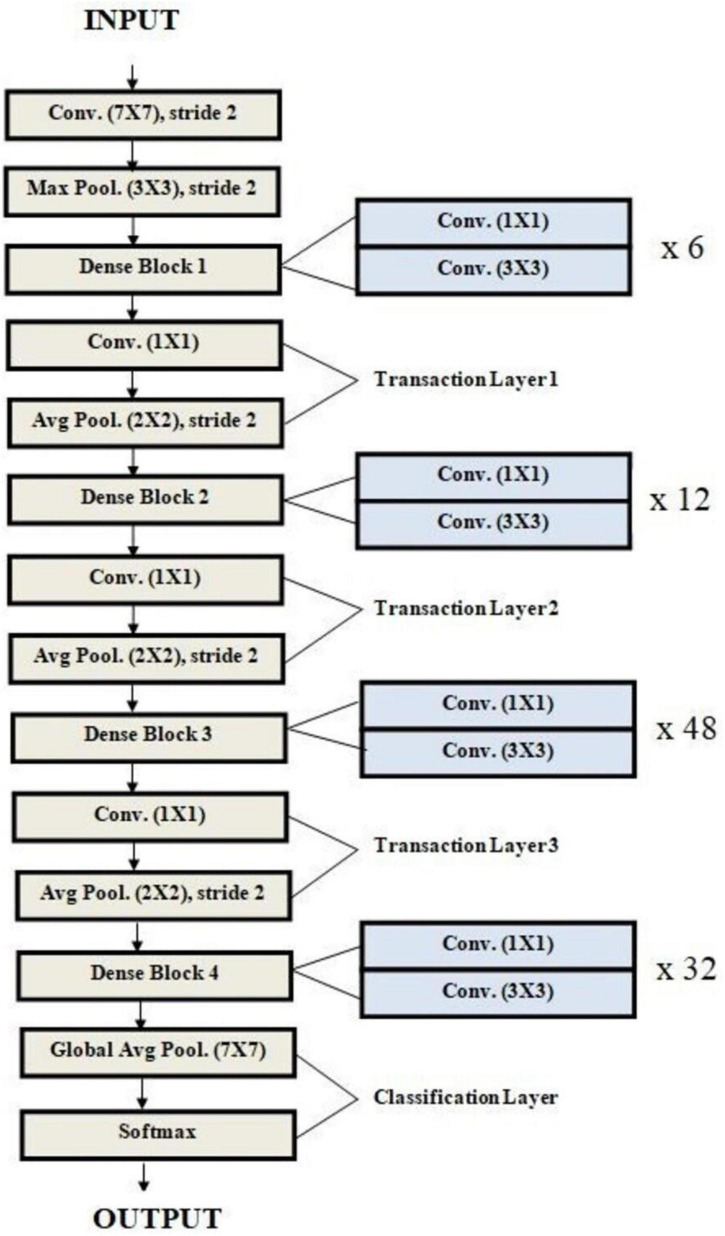
Architecture of DenseNet used for malaria prediction.

The proposed architecture is uniquely tailored for the malaria dataset, addressing challenges like feature variability and small dataset size. It is designed specifically for malaria cell images, balancing computational efficiency with predictive performance. Convolutional layers systematically capture features ranging from general (shapes) to specific (textures), enhancing accuracy. Dropout and L2 regularization prevent overfitting, ensuring the model generalizes well to unseen data. The compact architecture is computationally feasible, making it suitable for resource-constrained environments. The custom CNN’s modular design is visually represented in Figure 10, which outlines the sequential arrangement of layers, including convolutional, batch normalization, and dense layers. Additionally, Table 4 provides a detailed breakdown of each layer’s configuration, such as the number of filters, kernel sizes, activation functions, and regularization parameters. The hyperparameters that are fine-tuned to reduce error are listed in Table 5. Figure 11 illustrates the CNN-ML classifier diagram, highlighting the integration of the custom CNN architecture with various machine-learning algorithms in this study. Figure 12 shows the architecture of VGG16 used for Malaria Prediction. Figure 13 represents the architecture of VGG19 used for malaria prediction. Figure 14 shows the architecture of DenseNet used for Malaria Prediction. Overall, the custom CNN architecture effectively combines advanced feature extraction, robust regularization techniques, and efficient classification mechanisms. Its design ensures optimal performance for the malaria classification task, making it a viable solution for real-world applications in healthcare diagnostics.

**TABLE 4:** Transfer learning models with their coefficient measure.

Coefficients	Class/measure
Dimension	64 × 64
Sample quantity	42
Pooling	2 × 2
Epochs	105
Pre-initialized factors	ImageNet
Stimulation mechanism	SoftMax
Optimizer	Adam
Optimization coefficient	1e-04

**TABLE 5:** Coefficient measures that need to be fine-tuned.

Coefficients	Class/measures
Dimension	64 × 64
Sample quality	22
Iterations	98
Constraint	0.002
Omission	0.1
Stimulation mechanism	SoftMax
Optimizer	Adam
Optimization coefficient	1e-04

### 2.8 Experimental setup and evaluation metrics

The experiments for evaluating the malaria cell classification system were conducted in the Google Colab environment, leveraging its powerful computational resources, and streamlined setup. The hardware and software resources were; 69 gigabytes of available disk storage, 13 gigabytes of RAM, Nvidia Tesla T4 GPU for accelerated computations, 11.2 CUDA version which enabled GPU-optimized training via parallel processing, Python 3.7.10 was used for model development and analysis, and Matplotlib 3.2.2 package for plotting metrics and results. This configuration ensured a high-performance environment for the development, training, and evaluation of the proposed models, allowing rapid iterations and efficient handling of the large dataset.

The performance of the classification models was assessed using a suite of evaluation metrics designed to provide a comprehensive view of their effectiveness. These metrics include accuracy, precision, recall (sensitivity), F1 score, and specificity. Each metric measures a different aspect of model performance, particularly in the context of binary classification. Accuracy measures the proportion of correctly classified samples, encompassing both parasitized and uninfected cells and it is given as Equation 10. Recall evaluates the model’s ability to correctly identify positive cases (parasitized cells) and is given as Equation 11. Precision (Equation 12) quantifies the proportion of true positive predictions out of all positive predictions. Precision and Recall are crucial for understanding the trade-off between false positives and false negatives. In the context of this paper, a false positive (mislabeling an uninfected cell as parasitized) may lead to unnecessary treatment, and a false negative (failing to detect a parasitized cell) can have serious health consequences. F1 score represents the harmonic mean of precision and recall, balancing these two metrics, providing a balanced assessment by combining precision and recall into a single metric. It is given in Equation 13. The evaluation is also done using macro F1 score (Equation 14) and weighted average F1 score (Equation 15), where the former is the unweighted average of the F1 scores for each class, treating all classes equally and the latter takes the class imbalance into account by computing a weighted average of F1 scores. Specificity is used to measure the ability to correctly identify uninfected cells (negative cases). It is particularly important in this binary classification problem to ensure the accurate detection of uninfected cells, reducing unnecessary alarm. It is given in Equation 16.


A⁢c⁢c⁢u⁢r⁢a⁢c⁢y=TP+TN/TP+FP+TN+FN (12)

R⁢e⁢c⁢a⁢l⁢l⁢(T⁢P⁢R)=TP/TP+FN (13)

P⁢r⁢e⁢c⁢i⁢s⁢i⁢o⁢n⁢(P⁢P⁢V)=TP/TP+FN (14)

F⁢1⁢S⁢c⁢o⁢r⁢e= 2×Precision×Recall/Precision×Recall (15)

m⁢a⁢c⁢r⁢o⁢F⁢1=sum⁢of⁢all⁢the⁢F1⁢scores/Total⁢no.of⁢scores (16)

weighted⁢average⁢of⁢F1=sum⁢of⁢all⁢weighted⁢average⁢of (17)

F1⁢scores/Total⁢weighted⁢of⁢the⁢F1⁢Scores

S⁢p⁢e⁢c⁢i⁢f⁢i⁢c⁢i⁢t⁢y⁢(T⁢N⁢R)=TN/TN+FP (18)

By using these metrics, the models’ performance was rigorously evaluated, ensuring robustness and reliability for real-world deployment in malaria diagnosis systems and by combining robust preprocessing, advanced transfer learning, and ensemble techniques, the proposed system achieves superior malaria detection, paving the way for scalable applications in resource-constrained settings.

## 3 Results

**FIGURE 15:**
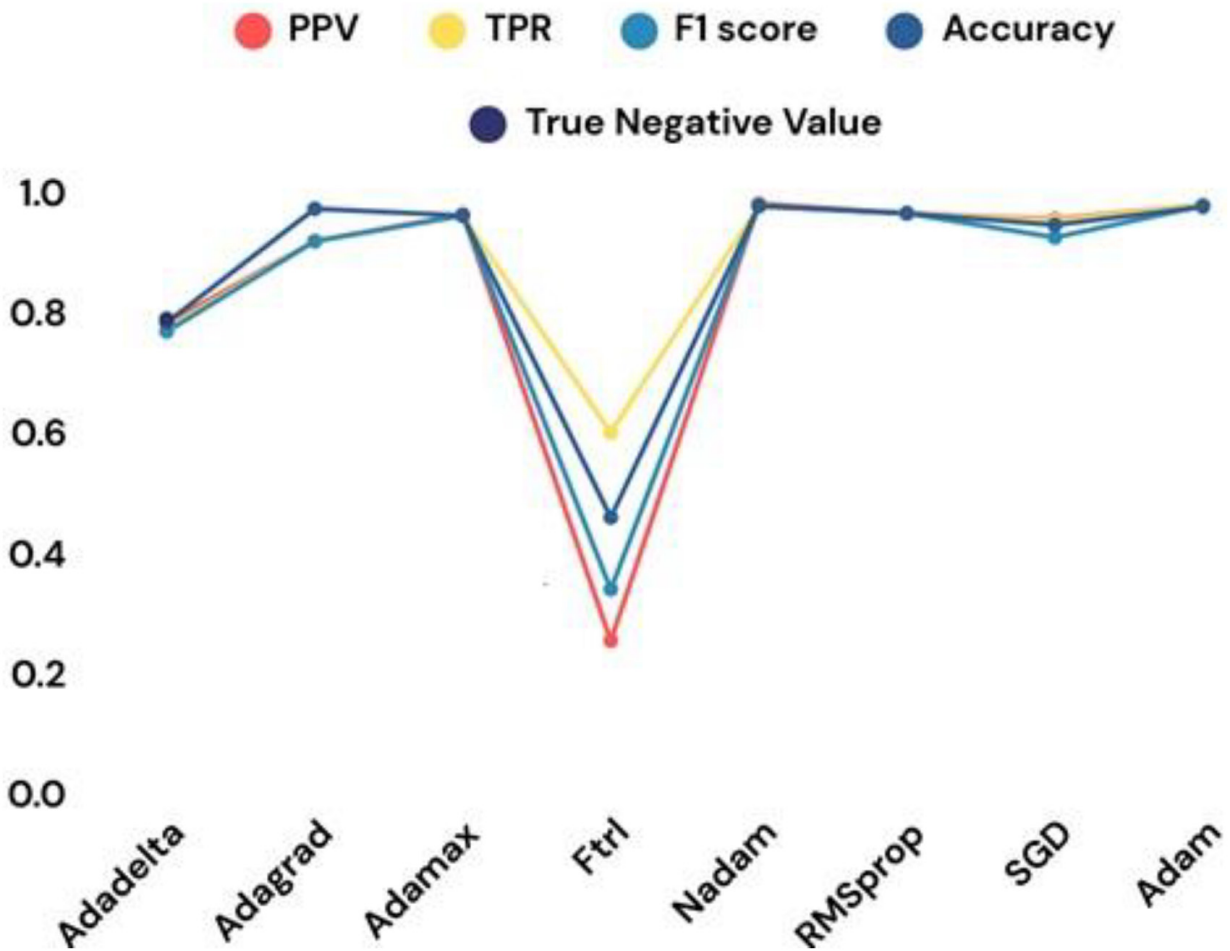
Performances at various optimizers.

**FIGURE 16:**
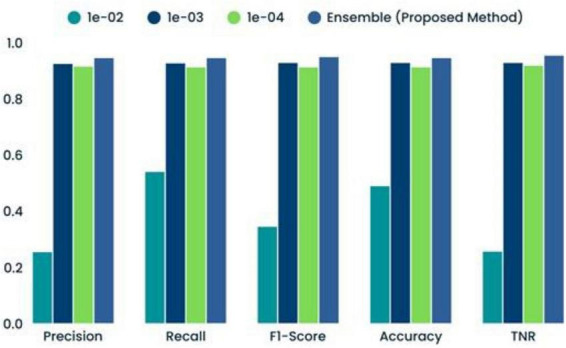
Comparison of several classifiers’ performances.

The experimental results of the study demonstrate the efficacy of the proposed methodologies in addressing the challenge of malaria detection through DL. The custom CNN achieved remarkable training and validation accuracies, highlighting its potential as a reliable feature extractor. The training accuracy reached 96.43%, and the validation accuracy was 96.56%. Without data augmentation, these values were 99.77% and 95.06%, respectively. The notable discrepancy between training and validation accuracy indicates signs of overfitting, where the model performs well on training data but exhibits diminished generalization on test data. Table 6 presents a detailed breakdown of the performance of the custom CNN across various optimizers, while Figure 15 graphically illustrates these results. The optimizers tested include Adadelta, Adagrad, Ftrl, SGD, Adam, and RMSprop, showcasing varying levels of effectiveness in reaching the global minimum loss. Outcomes for the modified CNN model’s efficacy at different learning rates are exhibited in Table 7 and Figure 16 is the graphical representation of the comparison of several classifiers’ performances.

**TABLE 6:** The Convolutional Neural Network (CNN) model’s results at various levels.

Techniques	PPV	TPR	F1 score	Accuracy	True negative value
Adadelta	0.7820	0.7740	0.7680	0.7830	0.7890
Adagrad	0.9180	0.9180	0.9179	0.9719	0.9810
Adamax	0.9625	0.9601	0.9605	0.9607	0.9652
Ftrl	0.2547	0.6001	0.3400	0.4593	0.4001
Nadam	0.9800	0.9765	0.9768	0.9764	0.9890
RMSprop	0.9643	0.9643	0.9643	0.9643	0.9643
SGD	0.9546	0.9522	0.9244	0.9452	0.9625
Adam	0.9789	0.9789	0.9768	0.9750	0.9810

**TABLE 7:** Comparison of several Convolutional Neural Network-machine learning (CNN-ML) classifiers’ performance results.

Optimization coefficient	Precision	Recall	F1-score	Accuracy	TNR
1e-02	0.2546	0.5400	0.3450	0.4895	0.2564
1e-03	0.9250	0.9268	0.9285	0.9285	0.9286
1e-04	0.9153	0.9128	0.9129	0.9129	0.9186
Ensemble (proposed method)	0.9456	0.9458	0.9489	0.9456	0.9543

**FIGURE 17:**
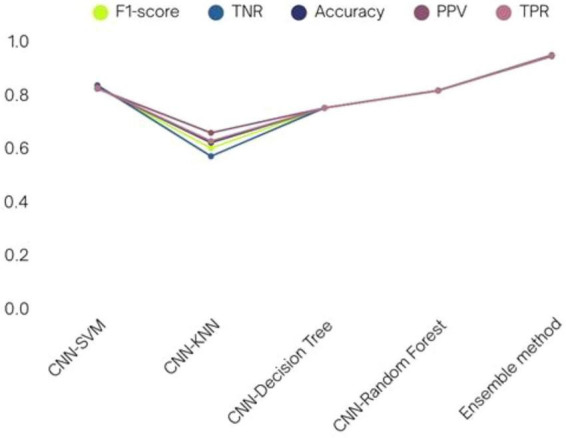
Various CNN-ML classifiers’ performance in terms of results.

**FIGURE 18:**
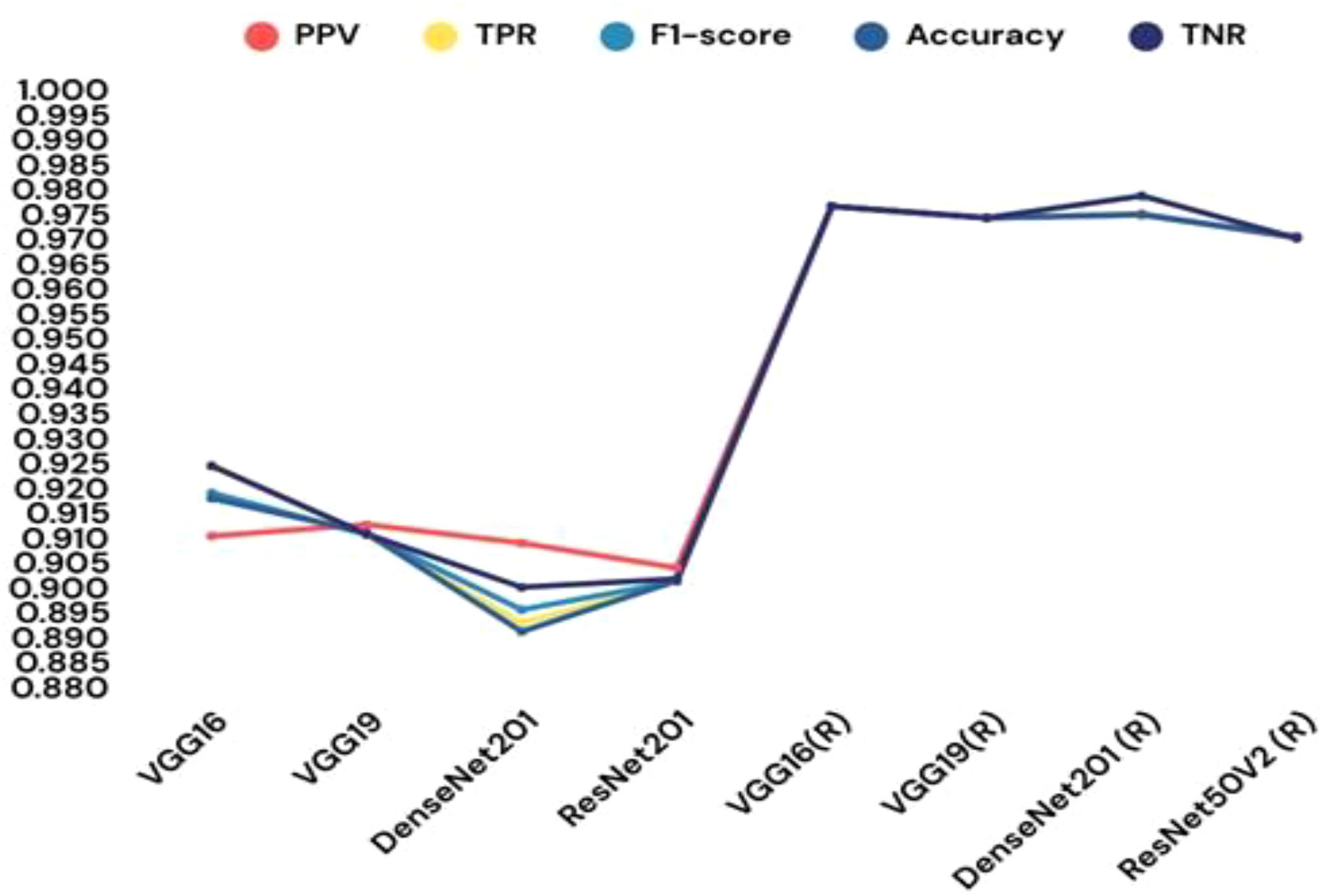
Different transfer learning models’ performance in terms of results.

Incorporating machine learning classifiers into the CNN architecture provided additional insights into the model’s adaptability. When the custom CNN served as a feature extractor, the SVM classifier achieved a commendable 81.67% test accuracy. A comparative analysis of various CNN-ML classifiers is presented in Table 8, with their respective performance outcomes visualized in Figure 17. These results demonstrate that the SVM-based combination was the most effective in leveraging CNN-extracted features for classification. The paper also explored transfer learning to assess the impact of pre-trained models on the malaria detection task. Retrained models, where all layers were fine-tuned, consistently outperformed their pre-trained counterparts. Among the tested architectures, VGG19 (retrained) exhibited the highest testing accuracy of 97.65%, outperforming other models. Table 9 systematically presents the performance outcomes of various transfer learning models, while Figure 18 provides a graphical comparison. These results underscore the importance of comprehensive training for achieving superior performance in novel domains.

**TABLE 8:** Various Convolutional Neural Network-machine learning (CNN-ML) classifiers’ performance in terms of results.

Model	PPV	TPR	F1-score	TNR	Accuracy
CNN-SVM	0.8214	0.8245	0.8266	0.8345	0.8247
CNN-KNN	0.6564	0.6255	0.5989	0.5689	0.6199
CNN-decision tree	0.7498	0.7498	0.7498	0.7498	0.7498
CNN-random forest	0.8145	0.8141	0.8143	0.8149	0.8148
Ensemble method (proposed method)	0.9432	0.9456	0.9453	0.9447	0.9472

**TABLE 9:** Different transfer learning models’ performance in terms of results.

Model	PPV	TPR	F1-score	Accuracy	TNR
VGG16	0.9102	0.9245	0.9189	0.9178	0.9243
VGG19	0.9125	0.9104	0.9105	0.9108	0.9106
DenseNet201	0.9088	0.8928	0.8954	0.8910	0.8999
ResNet201	0.9038	0.9010	0.9011	0.9012	0.9016
VGG16 (R)	0.9765	0.9765	0.9765	0.9765	0.9765
VGG19 (R)	0.9742	0.9742	0.9742	0.9742	0.9742
DenseNet201 (R)	0.9750	0.9748	0.9748	0.9748	0.9786
ResNet50V2 (R)	0.9701	0.9702	0.9703	0.9704	0.9701

**FIGURE 19:**
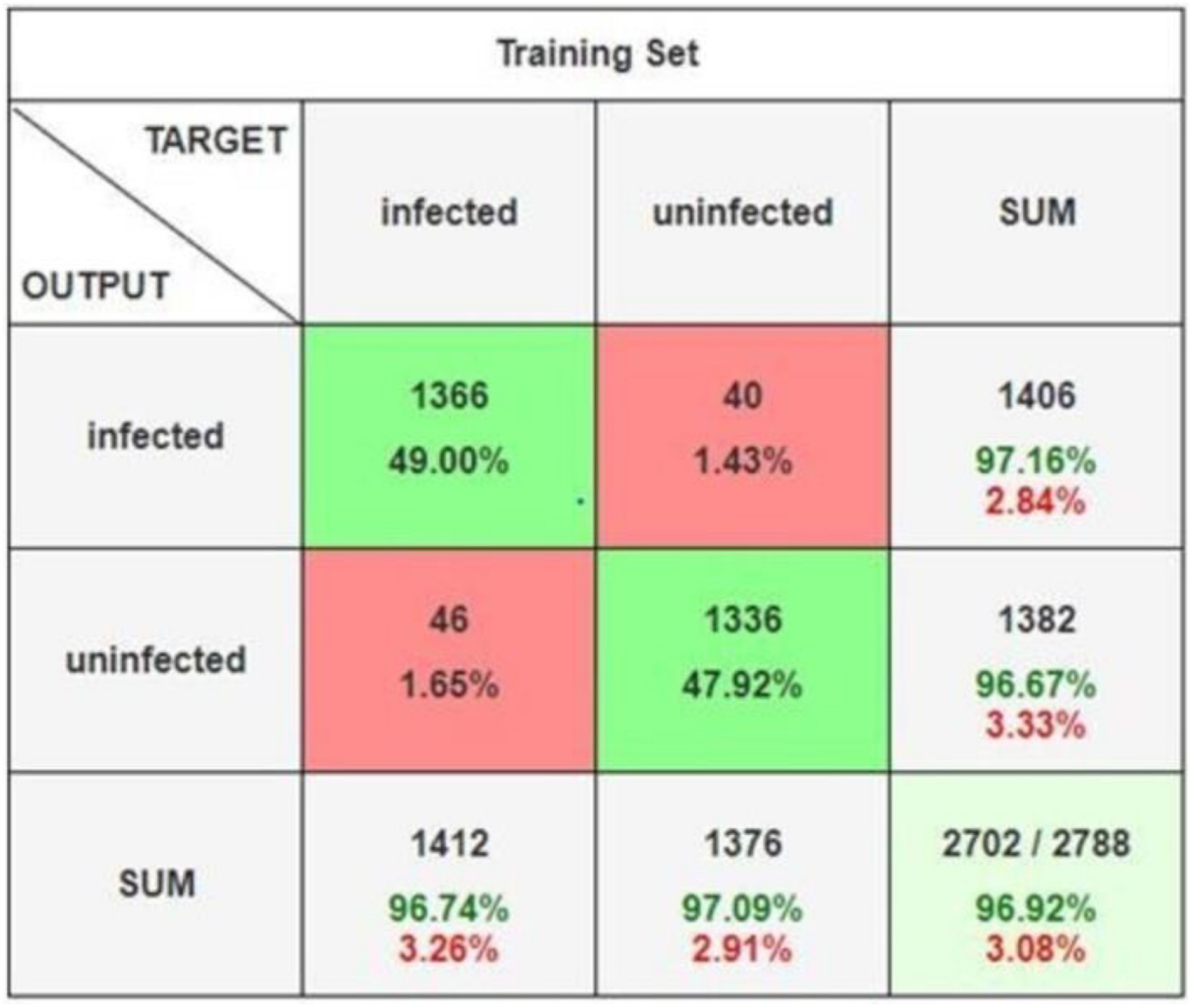
Confusion matrix.

**FIGURE 20:**
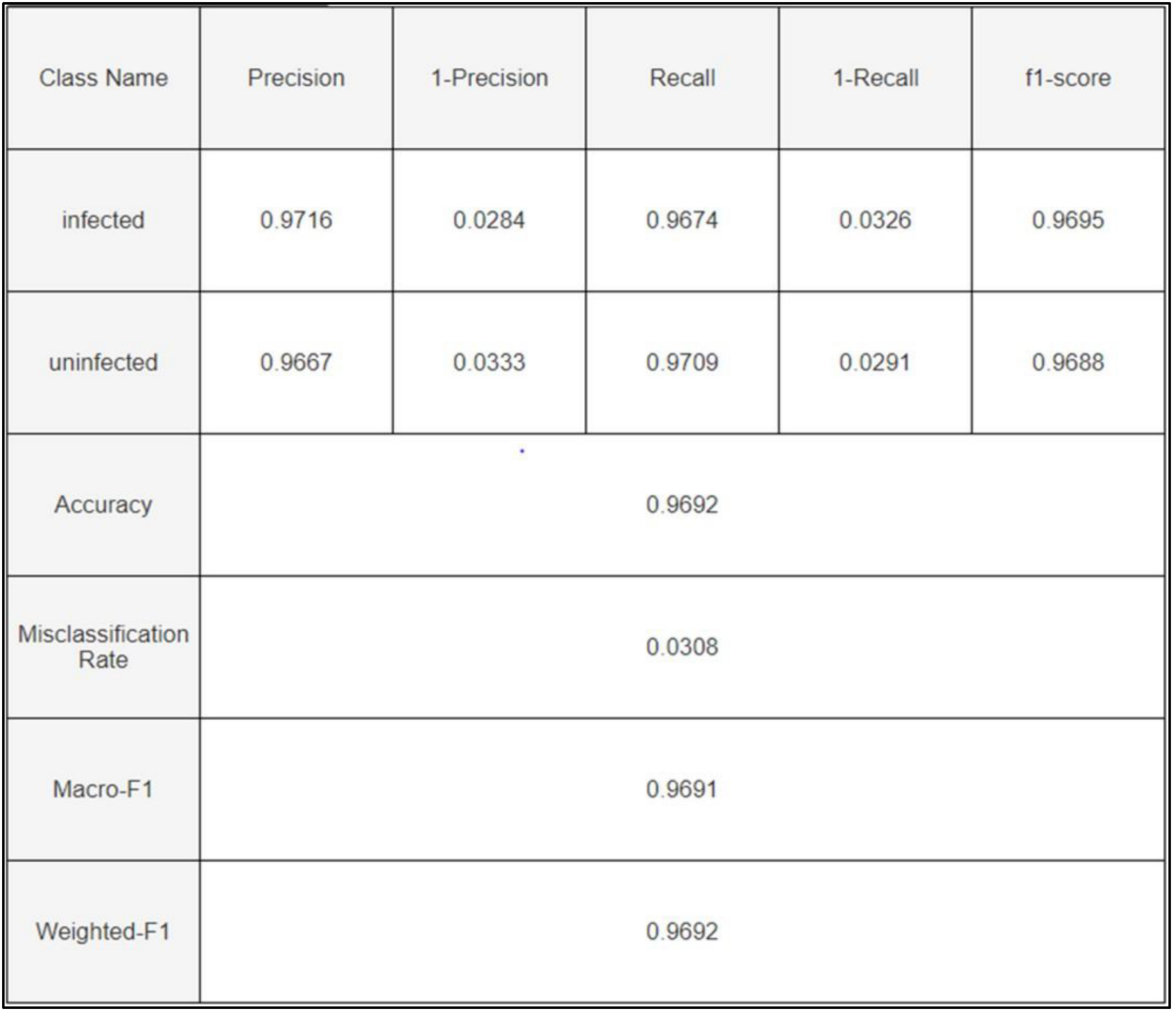
Detailed confusion matrix.

The ensemble learning approach, combining max voting and adaptive weighted averaging, further enhanced classification accuracy. For the parasitized class, 2,790 images were correctly classified, while 2,704 images from the uninfected class were accurately identified. The ensemble method achieved a testing accuracy of 97.93%, as reflected in the confusion matrices (Figures 18–20). These matrices indictae the robustness of the ensemble strategy in mitigating misclassifications. Table 10 provides a detailed summary of the results obtained from various ensemble learning techniques. The ensemble framework that combines hard voting approach ensured optimal outcomes by dynamically assigning appropriate weights to individual models. The proposed ensemble learning approach was compared to other deep learning models, demonstrating its superiority in malaria detection. The ensemble model achieved the highest testing precision of 97.93%, outperforming other models in terms of accuracy, precision, recall, and F1-score. Adaptive weighted averaging consistently improved validation accuracy by 0.5%–1.0% as compared to fixed-weight ensembles, demonstrating the efficacy of the dynamic weighting technique. Table 11 compares the performance of the proposed ensemble model against alternative methods, clearly illustrating its dominance in accuracy and robustness. The grid search gave DenseNet201 and VGG19 higher weights because of their superior individual performance, as Table 12 illustrates. The best validation accuracy of 97.93% was attained by the three-model ensemble (VGG16+VGG19+DenseNet201), which had ideal weights of 0.2, 0.4, and 0.4, respectively.

**TABLE 10:** Findings from several group education techniques.

Ensemble method	Model	Coefficients	TNR	PPV	TPR	F1-score	Accuracy
Dynamic weighted	VGG16 (R), VGG19 (R)	0.8, 0.5	0.9754	0.9789	0.9758	0.9758	0.9758
Average	VGG19 (R), DenseNet201 (R)	0.7, 0.6	0.9874	0.9874	0.9874	0.9874	0.9874
	VGG16 (R), DenseNet201(R)	0.5, 0.6	0.9761	0.9761	0.9761	0.9761	0.9761
	VGG16(R), VGG19 (R) and DenseNet201(R)	0.1, 0.8, 0.7	0.9788	0.9788	0.9788	0.9788	0.9788
Max voting	VGG16 (R), VGG19 (R) and DenseNet201 (R)	–	0.9782	0.9781	0.9780	0.9780	0.9780
Adaptive weighted average and max voting	VGG16 (R), VGG19 (R) and DenseNet201 (R)	–	0.9794	0.9794	0.9794	0.9794	0.9794

**TABLE 11:** Findings regarding alternative models juxtaposed with the proposed ensemble model.

Model	PPV	TNR	TPR	F1-score	Accuracy
Custom CNN	0.9728	0.9720	0.9721	0.9720	0.9720
VGG16 (R)	0.9765	0.9765	0.9765	0.9765	0.9765
CNN-SVM	0.8214	0.8245	0.8245	0.8266	0.8247
Ensemble (proposed method)	0.9793	0.9793	0.9793	0.9793	0.9793

**TABLE 12:** Optimal weights were determined for various ensemble model combinations (using grid search).

Ensemble combination	Search range	Optimal weights (model A/model B/model C)	Validation accuracy
VGG16 + VGG19	0.1–0.9 (step 0.1)	0.5/0.4	96.87
VGG16 + DenseNet201	0.1–0.9 (step 0.1)	0.3/0.6	97.12
VGG19 + DenseNet201	0.1–0.9 (step 0.1)	0.40/0.50	97.56
VGG16 + VGG19 + DenseNet201	0.1–0.9 (step 0.1)	0.2/0.4/0.4	97.93

Finally, the study compared the proposed approach with existing methodologies to evaluate its practical impact. The ensemble model outperformed previous works, particularly those using standalone CNN or transfer learning models. Table 13 presents a comparative evaluation, underscoring the ability of the ensemble model to detect malaria more precisely and effectively than existing methods. Overall, the proposed methodology demonstrates significant advancements in malaria detection, leveraging custom CNN architectures, transfer learning, and ensemble strategies. With an accuracy of 97.93%, the suggested ensemble outperformed both individual models and previous methods. Despite the slight (1–2%) absolute gain over earlier techniques, this improvement can have important therapeutic ramifications by lowering the likelihood of misdiagnosis, lowering false negatives, and increasing patient outcomes in malaria screening. By systematically addressing issues such as overfitting and leveraging the complementary strengths of multiple classifiers, the approach establishes a benchmark for future research in automated disease diagnosis. The superior accuracy, particularly of the ensemble model, highlights its potential for deployment in real-world healthcare applications.

**TABLE 13:** Comparing the results and performance of the proposed ensemble model with that of existing work.

Model	PPV	TPR	F1-score	TNR	Accuracy
Otsu segmentation, K-means clustering	0.9617	0.94	0.954	0.945	0.949
CNN	0.9547	0.9720	0.9720	0.9542	0.95
VGG16	0.9765	0.9765	0.9645	0.9654	0.9605
CNN	0.9689	0.9644	0.9689	0.9689	0.9689
VGG16	0.9709	0.9710	0.9699	0.9721	0.9780
VGG16	0.946	0.946	0.946	0.946	0.946
CNN	0.9781	0.9710	0.9748	0.9746	0.9749
**Ensemble model (proposed method)**	**0.9793**	**0.9793**	**0.9793**	**0.9763**	**0.9793**

## 4 Discussion

This paper introduces an ensemble learning-based deep neural network to identify malaria-causing parasites through microscopic red blood cell images. The suggested ensemble outperformed individual CNN and transfer learning models with an testing accuracy of 97.93% and demonstrated better classification robustness when compared to current benchmarks. This achievement is attributed to the integration of custom CNN architectures, transfer learning techniques, and adaptive ensemble strategies.

The proposed model’s accuracy significantly outperforms traditional single-network models and transfer learning-based approaches. For instance, while standalone custom CNN models and transfer learning methods such as VGG19 achieved commendable results, they were outmatched by the ensemble strategy, which mitigates individual model weaknesses. These results align with previous studies suggesting that ensemble techniques often provide enhanced robustness and generalizability by aggregating diverse model predictions. Furthermore, the integration of adaptive weighted averaging and max-voting mechanisms ensures optimal performance across a variety of test scenarios.

One critical challenge tackled in this research is overfitting, a common issue in deep learning models trained on biomedical datasets. Data augmentation and L2 regularization strategies effectively reduced overfitting, as evidenced by the reduced gap between training and validation accuracies. Such interventions resonate with prior studies that indicate the importance of pre-processing techniques and hyperparameter optimization in boosting model robustness for small or noisy datasets.

The ensemble model holds substantial promise for practical applications in biomedicine, particularly in resource-constrained settings where accurate malaria diagnosis is crucial. Microscopists could use this tool to expedite diagnosis while reducing the likelihood of human error. Moreover, the approach has the potential to be adapted for differentiating between various Plasmodium species, enabling more nuanced clinical decision-making. Future integration with mobile applications could further expand accessibility, empowering healthcare workers in remote or underserved areas. The frequency of misdiagnosed cases, particularly false negatives, can be significantly reduced with even a little increase in classification accuracy (1%–2%), which is essential for patient treatment. This demonstrates the suggested ensemble model’s potential clinical utility in supporting malaria diagnosis in practical settings. Unlike previous works that just used one CNN or transfer learning model, our ensemble approach incorporates a custom CNN along with many state-of-the-art architectures. Complementary feature learning is made possible by this integration, which also lowers the possibility of overfitting and produces more accurate categorization. This study’s uniqueness therefore resides in its ensemble structure, which goes beyond traditional single-model techniques.

Despite its successes, the paper acknowledges several limitations that impact the generalizability of the model like image quality variability, where fluctuations in the quality of microscopic images introduce inconsistencies in predictions, especially when working with diverse datasets. The reliance on specific datasets, such as the NIH dataset, may lead to biases that hinder the model’s performance on unfamiliar data or populations. Demographic or geographical biases inherent in the training data could distort predictions, particularly in global applications with diverse patient profiles. To mitigate these challenges, future research should focus on training the model using a broader spectrum of datasets, improving pre-processing pipelines, and developing techniques to handle variability in input data. The suggested framework delivers excellent diagnostic accuracy while providing practical benefit in clinical settings. It can help microscopists make better decisions by reducing workload and errors. Its lightweight design also makes it appropriate for mobile health platforms, which improves access in remote and underserved locations. Furthermore, interpretability approaches like Grad-CAM can provide visual explanations for forecasts, increasing transparency and clinician trust in the system.

Building on this work, future studies should aim to design new architectures tailored for multi-class classification tasks, such as differentiating between various Plasmodium species. Test the model on larger, more heterogeneous datasets to enhance its generalizability across different populations and contexts. Explore lightweight model designs suitable for deployment on mobile devices, making malaria diagnosis more accessible in remote regions. Investigate hybrid approaches combining machine learning and domain knowledge to refine model interpretability and usability in clinical settings. By addressing these directions, this line of research could significantly contribute to malaria elimination efforts, particularly in endemic regions.

## 5 Conclusion

The proposed research presents a novel ensemble learning-based deep neural network framework for malaria detection, demonstrating state-of-the-art performance with a testing accuracy of 97.93%. Through the strategic integration of custom CNN architectures, transfer learning models, and ensemble methods, the proposed system addresses key challenges such as overfitting, variability in image quality, and model generalization. The findings underscore the potential of machine learning, particularly ensemble approaches, to revolutionize malaria diagnosis. By leveraging robust pre-processing techniques, adaptive learning mechanisms, and diverse datasets, the model provides a reliable and efficient tool for assisting microscopists in clinical and field settings. The suggested ensemble model has the potential to assist in the clinical detection of malaria and exhibits good accuracy. However, as the present validation was limited to the NIH dataset and lacked hardware-level testing, immediate implementation in resource-constrained environments is still premature. To improve real-world applicability, future research will give priority to mobile deployment experiments and evaluation on external datasets. While this study focuses on binary classification, future work will include multi-class categorization of Plasmodium species. This clinically significant step addresses real-world diagnostic demands and improves the system’s translational utility.

Despite certain limitations, including data biases and inconsistencies in image quality, this research lays the foundation for further advancements in automated disease detection. In order to distinguish between Plasmodium species and test the model on external datasets, future research will concentrate on multi-class categorisation. The viability of implementation in clinical and resource-constrained outdoor settings will also be evaluated at the hardware level using smartphones and low-power embedded devices. Future efforts aimed at refining model generalization, exploring mobile applications, and expanding the dataset diversity will play a pivotal role in making these technologies universally applicable. The evaluation of the suggested ensemble model was restricted to images of Plasmodium falciparum and uninfected cells in a particular context, despite its good performance on the NIH dataset. As such, it is unclear whether it can be applied to other datasets or real-world samples with other staining techniques, imaging scenarios, or parasite species. More publicly accessible datasets should be evaluated in future research. Ultimately, the proposed system not only enhances diagnostic accuracy but also paves the way for scalable, data-driven solutions to malaria detection. This marks a significant step toward improving patient outcomes and supporting global health initiatives aimed at malaria eradication. This study is restricted to the NIH malaria dataset, which, despite its widespread use, has limited generalisability and may induce bias because of its controlled imaging circumstances. Future studies should investigate transfer learning between datasets acquired in various imaging conditions, address inter-laboratory variability using domain adaptation approaches, and replicate real-world changes in staining, illumination, and image quality using sophisticated augmentation techniques. This study’s reliance on the NIH dataset is a significant restriction, as it may limit the model’s applicability to other clinical or geographical circumstances. Cross-dataset testing involving images from various staining techniques, imaging devices, and patient groups should be included in future research. Furthermore, using domain adaptation approaches could improve the model’s robustness, ensuring that the suggested framework is more widely applicable in a variety of real-world scenarios. To improve the suggested model’s clinical applicability in a variety of resource-constrained contexts, these tactics must be put into practice.

## Data Availability

The original contributions presented in this study are included in this article/supplementary material, further inquiries can be directed to the corresponding authors.
